# Optical Resolution of Rimantadine

**DOI:** 10.3390/molecules24091828

**Published:** 2019-05-12

**Authors:** Jianlin Han, Ryosuke Takeda, Tatsunori Sato, Hiroki Moriwaki, Hidenori Abe, Kunisuke Izawa, Vadim A. Soloshonok

**Affiliations:** 1 Jiangsu Co-Innovation Center of Efficient Processing and Utilization of Forest Resources, College of Chemical Engineering, Nanjing Forestry University, Nanjing 210037, China; hanjl@njfu.edu.cn; 2 Hamari Chemicals Ltd., 1-4-29 Kunijima, Higashi-Yodogawa-ku, Osaka 533-0024, Japan; tatsunori-sato@hamari.co.jp (T.S.); hiroki-moriwaki@hamari.co.jp (H.M.); hidenori-abe@hamari.co.jp (H.A.); kunisuke-izawa@hamari.co.jp (K.I.); 3 Department of Organic Chemistry I, Faculty of Chemistry, University of the Basque Country UPV/EHU, Paseo Manuel Lardizábal 3, 20018 San Sebastián, Spain; 4 IKERBASQUE, Basque Foundation for Science, María Díaz de Haro 3, Plaza Bizkaia, 48013 Bilbao, Spain

**Keywords:** optical resolution, rimantadine, enantiomers, practicality, chiral acids and amines

## Abstract

This work discloses a new procedure for the resolution of commercially available racemic rimantadine hydrochloride to enantiomerically pure (*S*)-rimantadine using (*R*)-phenoxypropionic acid as a recyclable resolving reagent. Good chemical yields, operational ease, and low-cost structure underscore the preparative value of this method for the production of enantiomerically pure rimantadine for medicinal or synthetic studies.

## 1. Introduction

**Scheme 1:**
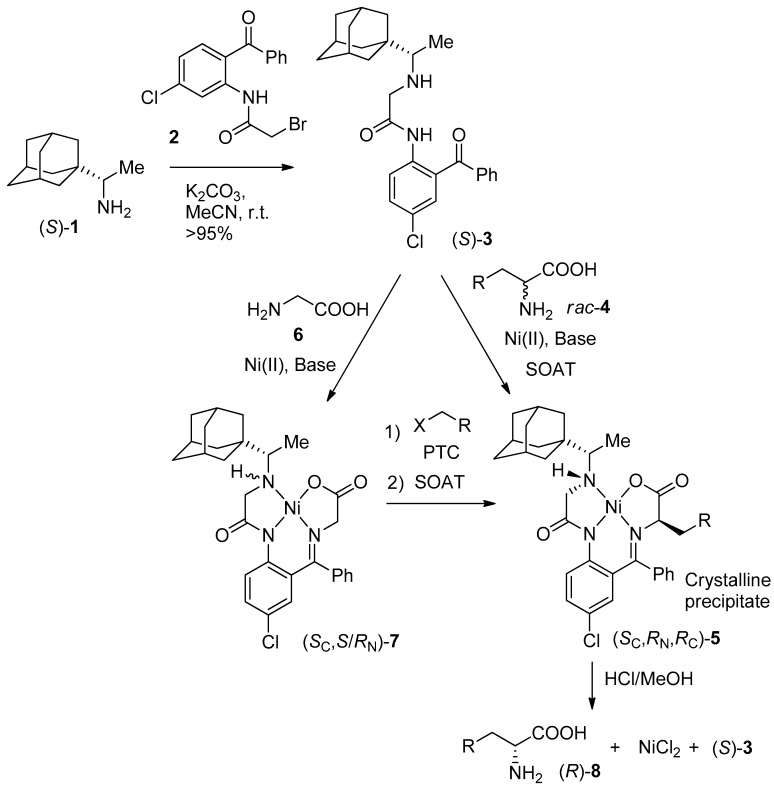
Application of recently designed (*S*)-rimantadine-derived ligand **3** for general asymmetric synthesis of α-amino acids (AAs) via Second-Order Asymmetric Transformation (SOAT) approach.

Unnatural, specially designed amino acids (AAs), commonly referred to as tailor-made AAs [1,2], play an increasingly important role in the development of modern pharmaceuticals and medicinal formulations. In fact, roughly about one-quarter of recently introduced drugs feature in their structure a residue of tailor-made AA [3,4,5,6]. Accordingly, the research in organic methodology for synthesis of tailor-made AAs is in extremely high demand in nearly every sector of the healthcare industry [7,8,9,10,11,12,13,14,15,16,17,18,19,20,21,22]. Our experience with synthesis of tailor-made AAs includes explorations in the areas of fluorine- [23,24,25], phosphorus- [26,27,28], and sulfur-containing AAs [29], as well as sterically constrained [30,31,32] and polyfunctional AA types [33,34,35]. More recently, we also became interested in the phenomenon of self-disproportionation of enantiomers [36,37,38], which is rather ubiquitous for AAs and their derivatives [39,40]. Nevertheless, our major activity in the field is concerned with the chemistry of AA Schiff base Ni(II) complexes as the general methodology for the asymmetric synthesis of tailor-made AAs [41,42,43,44]. In particular, using our modular approach for the design of chiral tridentate ligands [45,46], we recently introduced ligand (*S*)-**3** (Scheme 1) derived from enantiomerically pure rimantadine (*S*)-**1** and bromide **2**.

In the preliminary research [47,48], ligand (*S*)-**3** was found to perform potentially better, as compared with previous results [49,50,51], in the chemical dynamic kinetic resolution and (*S*)/(*R*)-interconversion of unprotected AAs. Specifically, due to the extreme bulkiness and lipophilicity of the rimantadine residue, intermediate complexes **5** of particular (*S*_C_,*R*_N_,*R*_C_) absolute configuration were easy to precipitate, allowing the realization of a second-order asymmetric transformation protocol for the preparation of tailor-made AAs **8**. It is, of course, understood that application of (*R*)-configured rimantadine will give rise to Ni(II) complexes **5** of (*R*_C_,*S*_N_,*S*_C_) absolute stereochemistry. Therefore, to continue our exploration of the synthetic potential of ligands of type **3**, we needed reliable access to enantiomerically pure (*S*)- and/or (*R*)-rimantadine. In this work, we report an optimized, economically sound procedure for optical resolution of commercially available racemic rimantadine with phenoxypropionic acid.

## 2. Results and Discussion

**Scheme 2:**
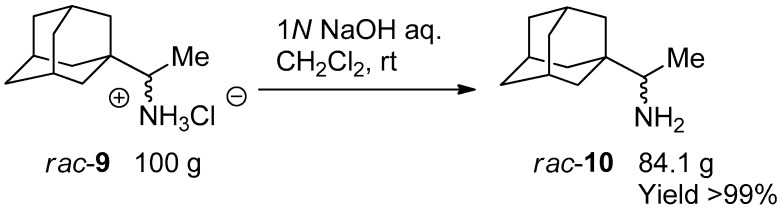
Transformation of rimantadine HCl salt **9** to free amine **10**.

Since its approval by the Food and Drug Administration (FDA) in 1994, rimantadine (α-methyl-1-adamantane-methalamine hydrochloride) (Scheme 2) is a marketed drug prescribed for the treatment of influenza virus A infection [52]. Despite some subtle differences in the binding properties of the (*S*)- and (*R*)-enantiomers **1** [53,54], in all its medical applications, rimantadine is used as a hydrochloric salt of the racemic form. Consequently, the research data for enantiomerically pure rimantadine and its commercial availability are quite limited. For example, the recent asymmetric synthesis of enantiomerically pure **1** [55] involves the reductive amination of the corresponding ketone using rather expensive Ellman’s chiral sulfinimide [56,57,58] as a chiral auxiliary. Accordingly, with the aim to develop an economically sound process, and drawing inspiration from the recent publications of optical resolutions of various chiral amines [59,60,61,62,63] and, in particular, patent data [64], we focused our attention on the optical resolution approach, the science of which is well understood and can be performed on an industrial scale [65,66,67,68].

At the outset, we needed to find a high-yield procedure for the preparation of racemic free-base rimantadine from the commercially available hydrochloric salt **9** (Scheme 2).

This goal was achieved using aqueous 1 N NaOH as a base in a biphasic system with dichloromethane, allowing for simultaneous extraction of free amine **10** into the organic layer. Target free rimantadine **10** was obtained in >99% yield and isolated simply by evaporating the organic solvent, before it was used for the consecutive experiments without additional purification.

**Scheme 3:**
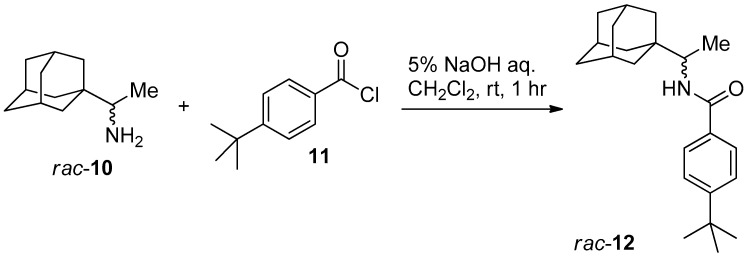
Preparation of derivative **12** as a standard for chiroptical analysis.

Our next task was to find a suitable standard compound for the convenient determination of an enantiomeric composition of rimantadine-derived diastereomeric salts. Following the literature [64], we prepared racemic amide **12** to have a reference for retention times of the corresponding (*S*)- and (*R*)-enantiomers. The target transformation was achieved via the treatment of free amine **10** with acyl chloride **11**, as presented in Scheme 3. The corresponding HPLC tests confirmed that *p*-*t*-Bu-benzoylated rimantadine **12** can perfectly serve the purpose of a reliable standard compound for routine HPLC analyses.

**Figure 1:**
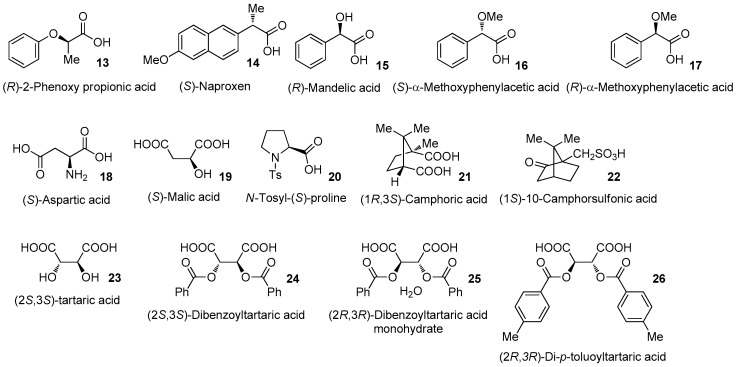
Structures of chiral carboxylic acids **13**–**26** used in this study.

With these results in hand, we proceeded with the main objective of this work, looking for a resolving reagent to achieve the optical resolution of racemic rimantadine **10**. For this task, we selected chiral acids **13**–**26** presented in Figure 1. All of these compounds are commercially available in both enantiomeric forms and were successfully used for the separation of various chiral amines [65,66,67,68].

The screening of resolving reagents **13**–**26** was conducted under standard conditions to compare the results and, thus, to find some promising candidates for an in-depth study. To this aim, we conducted a series of experiments using acetone as a solvent and using chiral acids **13**–**26** in 1.0, 0.5, and 0.25 molar stoichiometric ratios [65,66,67,68] relative to racemic rimantadine **10**. At the end of each experiment, the diastereomeric salt was treated with base, similar to the process presented in Scheme 2, to generate free rimantadine, following its conversion to the corresponding derivative **12** for the determination of the stereochemical outcome. The results are presented in Table 1. It should be noted that the absolute configuration of less soluble salts derived from the resolving reagent and rimantadine is always unpredictable [65,66,67,68]. Thus, in many cases (*S*)-configured acid gave preference for the (*S*)-enantiomer of rimantadine; however, in other cases, the relationships of the absolute configurations were opposite.

As one can see from Table 1, the results were generally very poor with the exception of (*R*)-2-phenoxy propionic acid **13**, (*S*)-naproxen **14**, (2*S*,3*S*)-dibenzoyltartaric acid **24**, and (2*R*,3*R*)-dibenzoyltartaric acid monohydrate **25**. Based on these data obtained, we selected (2*R*,3*R*)-dibenzoyltartaric acid **25** and (*R*)-2-phenoxypropionic acid **13** for more detailed study.

Firstly, we decided to explore (2*R*,3*R*)-dibenzoyl tartaric acid **25** as a resolving reagent due to its ready evaluability and reasonably low cost.

Table 2 summarizes a series of experiments conducted using 0.25 equivalents of resolving compound **25** in different organic solvents.

The data obtained were evaluated from the standpoint of both isolated yield of the crystalline precipitate of salt **31** and enantiomeric purity of the amine component in it. In our opinion, the best result was obtained in the case of the use of aqueous acetone as a solvent (entry 1) allowing isolation of salt **31** with excellent yield (46.8%) and reasonable enantiomeric purity (60.4% ee). Further attempts to improve this stereochemical outcome, using various amounts of water, concentrations, and temperature, were unfortunately without success. Therefore, we considered an option of additional purification of salt **31** obtained from the base experiment presented in entry 1.

As shown in Table 3, some success was achieved by crystallizing salt **31** from the same solvent (entry 1) or aqueous ethyl acetate (entry 2) and THF (entry 3).

However, in all cases, the results neither offered the enantiomeric composition nor the yields that can be considered as satisfactory for a sound practical procedure. For example, the additional, second crystallization from either solvent system did not bring the products much closer to enantiomerically pure (>99% ee) form. The best value achieved after the second crystallization was 94.8% ee, which was obtained using THF as a solvent.

Therefore, we decided to focus our attention on phenoxypropionic acid **13** as a resolution reagent. The search for a better-performing solvent brought about the same outcome as in the case of dibenzoyl tartaric acid **25**, pointing to aqueous (5%) acetone as the best solvent system. However, in sharp contrast to the latter, the option of additional purification via crystallization from a different solvent showed rather encouraging results (Table 4).

As shown in Table 4, we started with the mixing of racemic rimantadine **10** and (*R*)-phenoxypropionic acid **13** in aqueous (5%) acetone, resulting in the precipitation of diastereomeric salt **32**, which was isolated with 34.7% yield and showed 88.4% (*S*)-enantiomeric excess of the amine residue in it. Thus, obtained product **32** was next crystallized from aqueous ethyl acetate allowing precipitation of 87.3% of the original material **32** with noticeably increased enantiomeric purity (98.7% ee) of the constituent residue of rimantadine. One more crystallization procedure was performed using the same solvent system, affording salt **32** with 92% yield and enantiomeric purity of the target amine exceeding 99% ee. The overall yield of this three-step procedure was 28.1%, which we considered as adequate to provide reliable access to enantiomerically pure rimantadine.

**Scheme 4:**
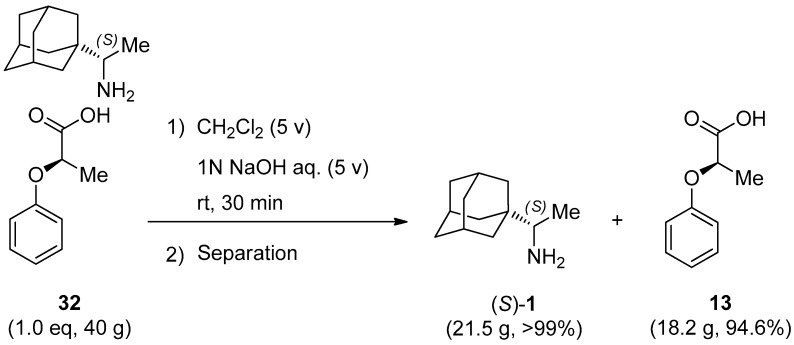
Isolation of enantiomerically pure (99.7% ee) rimantadine (*S*)-**1** from salt **32**.

Finally, we needed to develop a protocol for the isolation of free rimantadine (*S*)-**1** from diastereomerically pure salt **32**. As one can see from Scheme 4, salt **32** was treated with NaOH in a biphasic system with dichloromethane, allowing the extraction of the released rimantadine (*S*)-**1** in the organic layer. Expectedly, this simple procedure afforded target free rimantadine (*S*)-**1** with nearly quantitative chemical yield and of uncompromised (99.7% ee) enantiomeric purity.

Importantly, the resolving reagent (*R*)-**13** was also recovered with excellent chemical yield. It should be noted that recycling of chiral acid (*R*)-**13** is very important for the overall low-cost preparation of enantiomerically pure rimantadine (*S*)-**1**.

## 3. Materials and Methods

### 3.1. General Methods

All reagents and solvents were used as received. Reactions were monitored by thin-layer chromatography on Merck silica gel 60-F_254_ coated 0.25-mm plates, detected by ultraviolet (UV). Flash chromatography was performed with the indicated solvents on silica gel (particle size 0.064–0.210 mm). Yields reported are for isolated, spectroscopically pure compounds. HPLC was performed on a SHIMADZU LC-2010CHT chromatography system and a CLASS-VPTM analysis data system. ^1^HNMR spectra were recorded on a Brüker AVANCE III-400 spectrometer. Chemical shifts are given in ppm (*d*), referenced to tetramethylsilane (TMS). The letters s, d, t, q, m, and br stand for singlet, doublet, triplet, quartet, multiplet, and broad, respectively. Melting points were recorded on a Mettler Toledo MP70 Melting Point System and are not corrected.

### 3.2. Transformation of Rimantadine HCl Salt **9** to Free Amine **10**

To a mixture of racemic rimantadine HCl **9** (100 g, 463.5 mmol) in CH_2_Cl_2_ (1000 mL), 1 N NaOH (1000 mL) was added. The reaction mixture was stirred at room temperature for 30 min. The resultant mixture was separated. The organic layer was washed with 1 N NaOH (500 mL) and water (3 × 500 mL) then dried over Na_2_SO_4_. The organic layer was evaporated to afford racemic rimantadine **10** as a white residue (84.1 g, yield: >99%).

^1^H NMR (200 MHz, CDCl_3_): δ 0.97 (d, *J* = 6.6 Hz, 3H), 1.46−1.53 (br, 6H), 1.56–1.79 (m, 6H), 1.92–2.04 (m, 3H), 2.40 (q, *J* = 6.6 Hz, 1H). ^13^C NMR (50 MHz, CDCl_3_): δ 16.9, 28.5, 35.8, 37.3, 38.1, 55.8.

### 3.3. General Procedure for Resolution

To the solution of racemic rimantadine **10** in acetone with 5% H_2_O, resolving reagent **13−26** was added in acetone with 5% H_2_O at 50 °C. The amount of solvent was adjusted to 12 volumes. The mixture was stirred for 30 min at 50 °C, then cooled to room temperature and stirred for 24 h to form the corresponding salt. The salt was filtered, washed with acetone with 5% H_2_O, and dried under vacuum (<0.5 mmHg) at room temperature (rt). The solvent for crystallization is indicated in Table 1, Table 2, Table 3 and Table 4 for each particular experiment and salt compound (Supplementary Materials).

#### 3.3.1. Rimantadine (**10**) (*R*)-2-Phenoxy Propionic Acid (**13**) 0.5 Eq. Salt (99.7% ee)

Molecular weight (Mw): 51.1 g, 92.9% yield, 99.7% ee (*S*) from 55.0 g, 98.7% ee (*S*) salt, melting point (mp): 167–171 °C. ^1^H NMR (400 MHz, CD_3_OD): δ = 7.15–7.29 (m, 2H), 6.80–6.92 (m, 3H), 4.40 (q, *J* = 6.7 Hz, 1H), 2.75 (q, *J* = 6.6 Hz, 1H), 1.96–2.09 (m, 3H), 1.55–1.86 (m, 12H), 1.42–1.52 (m, 4H), 1.15 (d, *J* = 6.6 Hz, 3H).

#### 3.3.2. Rimantadine (**10**) (*S*)-Naproxen (**14**) 0.5 Eq. Salt

Mw: 0.51 g, 44.8% yield, 35.3% ee (*R*), mp: 164–167 °C. ^1^H NMR (400 MHz, CD_3_OD): δ = 7.61–7.72 (m, 3H), 7.45–7.55 (m, 1H), 7.15–7.20 (m, 1H), 7.02–7.11 (m, 1H), 3.85 (s, 3H), 3.61–3.79 (m, 1H), 3.65–3.85 (m, 1H), 1.95–2.05 (m, 3H), 1.50–1.85 (m, 12H), 1.45–1.49 (m, 3H), 1.11 (d, *J* = 6.6 Hz, 3H).

#### 3.3.3. Rimantadine (**10**) (*R*)-Mandelic Acid (**15**) 0.5 Eq. Salt

Mw: 0.39 g, 42.0% yield, 7.32% ee (*S*), mp: 154–156 °C. ^1^H NMR (400 MHz, CD_3_OD): δ = 7.40–7.55 (m, 2H), 7.16–7.39 (m, 3H), 4.85 (s, 1H), 2.76 (q, *J* = 6.7 Hz, 1H), 1.97–2.09 (m, 3H), 1.42–1.89 (m, 13H), 1.13 (d, *J* = 6.7 Hz, 3H).

#### 3.3.4. Rimantadine (**10**) (*S*)-α-Methoxyphenylacetic Acid (**16**) 0.5 Eq. Salt

Mw: 0.35 g, 36.1% yield, 1.34% ee (*S*), mp: 178–181 °C. ^1^H NMR (400 MHz, CD_3_OD): δ = 7.45–7.55 (m, 2H), 7.21–7.37 (m, 3H), 4.52 (s, 1H), 3.36 (s, 3H), 2.75 (q, *J* = 6.6 Hz, 1H), 1.95–2.06 (m, 3H), 1.42–1.89 (m, 13H), 1.13 (d, *J* = 6.6 Hz, 3H).

#### 3.3.5. Rimantadine (**10**) (*R*)-α-Methoxyphenylacetic Acid (**17**) 0.5 Eq. Salt

Mw: 0.39 g, 40.1% yield, 0.08% ee (*S*), mp: 177–181 °C. ^1^H NMR (400 MHz, CD_3_OD): δ = 7.45–7.55 (m, 2H), 7.20–7.39 (m, 3H), 4.55 (s, 1H), 3.31 (s, 3H), 2.79 (q, *J* = 6.7 Hz, 1H), 1.95–2.09 (m, 3H), 1.49–1.85 (m, 13H), 1.15 (d, *J* = 6.6 Hz, 3H).

#### 3.3.6. Rimantadine (**10**) (*S*)-Aspartic Acid (**18**) 0.5 Eq. Salt

Mw: 0.36 g, 52.0% yield, 7.44% ee (*S*), mp: 205–208 °C. ^1^H NMR (400 MHz, CD_3_OD): δ = 4.69–4.79 (m, 1H), 2.75–2.93 (m, 2H), 2.49–2.63 (m, 1H), 1.98–2.09 (m, 3H), 1.45–1.90 (m, 13H), 1.18 (d, *J* = 6.6 Hz, 3H).

#### 3.3.7. Rimantadine (**10**) (*S*)-Aspartic acid (**18**) 0.25 Eq. Salt (1:1 Salt)

Mw: 0.17 g, 24.6% yield, 7.20% ee (*S*), mp: 207–209 °C. ^1^H NMR (400 MHz, CD_3_OD): δ = 4.67–4.78 (m, 1H), 2.76–2.91 (m, 2H), 2.48–2.61 (m, 1H), 1.98–2.09 (m, 3H), 1.50–1.90 (m, 13H), 1.15 (d, *J* = 6.7 Hz, 3H).

#### 3.3.8. Rimantadine (**10**) (*S*)-Malic Acid (**19**) 1.0 Eq. Salt

Mw: 0.52 g, 59.2% yield, 0.20% ee (*S*), mp: 206–209 °C. ^1^H NMR (400 MHz, CD_3_OD): δ = 4.21–4.31 (m, 1H), 2.80–2.92 (m, 2H), 2.65–2.79 (m, 1H), 2.35–2.51 (m, 1H), 1.98–2.12 (m, 6H), 1.49–1.89 (m, 27H), 1.18 (d, *J* = 6.7 Hz, 6H).

#### 3.3.9. Rimantadine (**10**) (*S*)-Malic Acid (**19**) 0.25 Eq. Salt

Mp: 207–211 °C. ^1^H NMR (400 MHz, CD_3_OD): δ = 4.21–4.31 (m, 1H), 2.78–2.92 (m, 2H), 2.65–2.79 (m, 1H), 2.39–2.51 (m, 1H), 1.97–2.10 (m, 6H), 1.51–1.88 (m, 26H), 1.18 (d, *J* = 6.7 Hz, 6H).

#### 3.3.10. Rimantadine (**10**) *N*-Tosyl-(*S*)-proline (**20**) 0.5 Eq. Salt

Mw: 0.08 g, 6.4% yield, 95.46% ee (*R*), mp: 178–181 °C. ^1^H NMR (400 MHz, CD_3_OD): δ = 7.72–7.81 (m, 2H), 3.95–4.05 (m, 1H), 3.45–3.55 (m, 2H), 3.15–3.25 (m, 1H), 2.80–2.94 (m, 1H), 2.53 (s, 3H), 2.65–2.79 (m, 1H), 2.00–2.11 (m, 3H), 1.59–1.89 (m, 15H), 1.19 (d, *J* = 6.6 Hz, 3H).

#### 3.3.11. Rimantadine (**10**) (1*R*,3*S*)-Camphoric Acid (**21**) 0.5 Eq. Salt

Mw: 0.78 g, 100% yield, 0.72% ee (*S*), mp: 180–182 °C. ^1^H NMR (400 MHz, CD_3_OD): δ = 2.70–2.90 (m, 1H), 2.42–2.70 (m, 1H), 1.98–2.11 (m, 3H), 1.60–1.88 (m, 14H), 1.45 (s, 1H), 1.35 (s, 3H), 1.18 (d, *J* = 6.6 Hz, 3H), 0.90 (s, 2H).

#### 3.3.12. Rimantadine (**10**) (1*R*,3*S*)-Camphoric Acid (**21**) 0.25 Eq. Salt

Mw: 0.13 g, 16.8% yield, 0.38% ee (*S*), mp: 182–186 °C. ^1^H NMR (400 MHz, CD_3_OD): δ = 2.70–2.85 (m, 1H), 2.52–2.70 (m, 1H), 1.97–2.12 (m, 3H), 1.55–1.88 (m, 12H), 1.38 (s, 1H), 1.14–1.22 (m, 6H), 0.90 (s, 1H).

#### 3.3.13. Rimantadine (**10**) (1*S*)-10-Camphorsulforic Acid (**22**) 1.0 Eq. Salt

Mw: 0.81 g, 70.9% yield, 5.22% ee (*R*), mp: 204–208 °C. ^1^H NMR (400 MHz, CD_3_OD): δ = 3.31–3.41 (m, 2H), 2.82 (q, *J* = 6.5 Hz, 1H), 2.60–2.80 (m, 2H), 2.28–2.49 (m, 1H), 2.00–2.18 (m, 5H), 1.40–1.90 (m, 16H), 1.20 (d *J* = 6.5 Hz, 3H), 1.15 (s, 3H), 0.89 (s, 3H).

#### 3.3.14. Rimantadine (**10**) (2*S*,3*S*)-Tartaric Acid (**23**) 0.5 Eq. Salt

Mw: 0.73 g, 103% yield, 1.38% ee (*S*), mp: 220–221 °C. ^1^H NMR (400 MHz, CD_3_OD): δ = 4.30 (s, 1H), 2.80 (q, *J* = 6.5 Hz, 1H), 1.96–2.18 (m, 3H), 1.45–1.89 (m, 12H), 1.15 (d, *J* = 6.5 Hz, 3H).

#### 3.3.15. Rimantadine (**10**) (2*S*,3*S*)-Tartaric Acid (**23**) 0.25 Eq. Salt

Mw: 0.35 g, 49.0%, 23.24% ee (*S*), mp: 217–219 °C. ^1^H NMR (400 MHz, CD_3_OD): δ = 4.30 (s, 1H), 2.81 (q, *J* = 6.6 Hz, 1H), 1.97–2.08 (m, 3H), 1.44–1.88 (m, 13H), 1.12 (d, *J* = 6.5 Hz, 3H).

#### 3.3.16. Rimantadine (**10**) (2*S*,3*S*)-Dibenzoyltartaric Acid (**24**) 0.5 Eq. Salt (2:1 Salt)

Mw: 0.87 g, 86.6% yield, 16.38% ee (*R*), mp: 168–171 °C. ^1^H NMR (400 MHz, CD_3_OD): δ = 8.15–8.22 (m, 2H), 7.50–7.75 (m, 3H), 5.89 (s, 1), 2.72 (q, *J* = 6.6 Hz, 1H), 1.91–2.08 (m, 3H), 1.42–1.89 (m, 12H), 1.11 (d, *J* = 6.5 Hz, 3H).

#### 3.3.17. Rimantadine (**10**) (2*S*,3*S*)-Dibenzoyltartaric Acid (**24**) 0.25 Eq. Salt

Mw: 0.48 g, 48.4% yield, 62.72% ee (*R*), mp: 178–179 °C. ^1^H NMR (400 MHz, CD_3_OD): δ = 8.12–8.22 (m, 2H), 7.40–7.75 (m, 3H), 5.86 (s, 1), 2.72 (q, *J* = 6.5 Hz, 1H), 1.92–2.08 (m, 3H), 1.40–1.85 (m, 12H), 1.10 (d, *J* = 6.5 Hz, 3H).

#### 3.3.18. Rimantadine (**10**) (2*R*,3*R*)-Dibenzoyltartaric Acid Monohydrate (**25**) 0.25 Eq. Salt (94.8% ee)

Mw: 0.242 g, 48.4% yield, 94.8% ee (*S*) from 0.50 g, 67.0% ee (*S*) salt, mp: 177–179 °C. ^1^H NMR (400 MHz, CD_3_OD): δ = 8.13–8.22 (m, 2H), 7.50–7.77 (m, 3H), 5.87 (s, 1), 2.72 (q, *J* = 6.5 Hz, 1H), 1.92–2.12 (m, 3H), 1.40–1.85 (m, 12H), 1.11 (d, *J* = 6.5 Hz, 3H).

#### 3.3.19. Rimantadine (**10**) (2*R*,3*R*)-Di-*p*-toluoyltartaric Acid (**26**) 1.0 Eq. Salt

Mw: 1.612 g, 102% yield, 0.38% ee (*R*), mp: 215–216 °C. ^1^H NMR (400 MHz, CD_3_OD): δ = 7.97–8.05 (m, 4H), 7.25–7.35 (m, 4H), 5.87 (s, 1), 2.75–2.90 (m, 1H), 2.40 (s, 6H), 1.95–2.08 (m, 3H), 1.40–1.86 (m, 12H), 1.15 (d, *J* = 6.5 Hz, 3H).

#### 3.3.20. Rimantadine (**10**) (2*R*,3*R*)-Di-*p*-toluoyltartaric Acid (**26**) 0.25 Eq. Salt

Mw: 0.53 g, 50.6% yield, 14.12% ee (*S*), mp: 177–180 °C. ^1^H NMR (400 MHz, CD_3_OD): δ = 8.01–8.09 (m, 2H), 7.20–7.31 (m, 2H), 5.82 (s, 1), 2.70–2.88 (m, 1H), 2.40 (s, 3H), 1.90–2.06 (m, 3H), 1.40–1.86 (m, 13H), 1.10 (d, *J* = 6.5 Hz, 3H).

### 3.4. Isolation of Enantiomerically Pure (99.7% ee) Rimantadine (S)-**1** from Salt **32**

To a mixture of salt **32** (40 g, 116 mmol) in CH_2_Cl_2_ (200 mL), 1 N NaOH (200 mL) was added. The reaction mixture was stirred at room temperature for 30 min. The resultant mixture was separated. Organic layer was washed with 1 N NaOH (100 mL) and water (2 × 80 mL), then dried over Na_2_SO_4_. The organic layer was evaporated and dried under vacuum to afford enantiomerically pure rimantadine (*S*)-**1** as a white solid (21.5 g, yield: >99%).

## 4. Conclusions

In summary, we developed a new procedure for the resolution of commercially available racemic rimantadine hydrochloride to enantiomerically pure (*S*)-rimantadine free base. The resolving reagent (*R*)-phenoxypropionic acid, used in this method, can be conveniently recycled and reused for continuous preparation of the target chiral amine. Reasonably good chemical yields and operational ease of all transformations, coupled with low overall cost, bode well for its synthetic value for the preparation of enantiomerically pure rimantadine.

## Figures and Tables

**Table 1:**
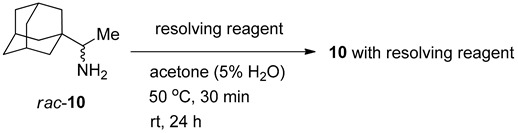
Screening resolving reagents **13**–**26** under standard conditions.

	Resolving Reagent	Equivalent of Resolving Reagent ^2,3^		
	in Acetone (5% H_2_O) (12 v) ^1^	1.0 eq	0.5 eq	0.25 eq
**13**	(*R*)-2-Phenoxy propionic acid	-	0.48 g, 35.5%	-
			88.0% ee (*S*)	
**14**	(*S*)-Naproxen	1.04 g, 91.2%	0.51 g, 44.8%	-
		11.0% ee^4^ (*R*)	35.3% ee (*R*)	
**15**	(*R*)-Mandelic acid	0.86 g, 93.3%	0.39 g, 42.0%	-
		2.82% ee (*S*)	7.32% ee (*S*)	
**16**	(*S*)-α-Methoxyphenylacetic acid	0.83 g, 86.1%	0.35 g, 36.1%	-
		0.88% ee (*S*)	1.34% ee (*S*)	
**17**	(*R*)-α-Methoxyphenylacetic acid	0.84 g, 86.7%	0.39 g, 40.1%	-
		0.08% ee (*S*)	0.08% ee (*S*)	
**18**	(*S*)-Aspartic acid	0.80 g, 91.3%	0.36 g, 52.0%	0.17 g, 24.6%
		4.86% ee (*S*)	7.44% ee (*S*)	7.20% ee (*S*)
**19**	(*S*)-Malic acid	0.52 g, 59.2%	-	0.35 g, 50.6%
		0.20% ee (*S*)		0.26% ee (*S*)
**20**	*N*-Tosyl-(*S*)-proline	0.35 g, 28.3%	0.08 g, 6.4%	-
		94.96% ee (*R*)	95.46% ee (*R*)	
**21**	(1*R*,3*S*)-Camphoric acid	1.03 g, 97.5%	0.78 g, >98%	0.13 g, 16.8%
		1.36% ee (*S*)	0.72% ee (*S*)	0.38% ee (*S*)
**22**	(1*S*)-10-Camphorsulforic acid	0.81 g, 70.9%	-	-
		5.22% ee (*R*)		
**23**	(2*S*,3*S*)-Tartaric acid	0.92 g, 95.0%	0.73 g, >98%	0.35 g, 49.0%
		0.08% ee (*S*)	1.38% ee (*S*)	23.24% ee (*S*)
**24**	(2*S*,3*S*)-Dibenzoyltartaric acid	1.41 g, 94.2%	0.87 g, 86.6%	0.48 g, 48.4%
		0.28% ee (*S*)	16.38% ee (*R*)	62.72% ee (*R*)
**25**	(2*R*,3*R*)-Dibenzoyltartaric acid monohydrate	-	-	0.47 g, 46.8%
				**60.44% ee (*S*)**
**26**	(2*R*,3*R*)-Di-*p*-toluoyltartaric acid	1.612 g, >98%		0.53 g, 50.6%
		0.38% ee (*R*)		14.12% ee (*S*)

^1^ Total amount of solvent was adjusted to 12 volumes. ^2^ Yield was calculated based on the molar number of amine obtained as the salt. ^3^ Each salt was analyzed via in situ preparation of free amine and transformation to **12** followed by chiral HPLC analysis. ^4^ ee: enantiomeric excess

**Table 2:**
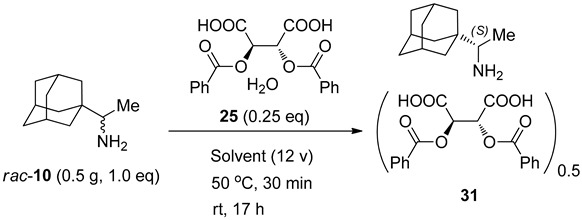
The use of 0.25 equivalents of (2*R*,3*R*)-dibenzoyltartaric acid **25** in various solvents.

Entry	Solvent (12 v)	Results ^1^
1	Acetone (5% H_2_O)	0.47 g, 46.8%
		60.4% ee (*S*)
2	THF^2^	0.32 g, 31.8%
		36.8% ee (*S*)
3	IPE^2^	0.52 g, 52.4%
		36.2% ee (*S*)
4	Acetone	0.51 g, 51.1%
		32.9% ee (*S*)
5	EtOAc	0.50 g, 50.4%
		28.4% ee (*S*)
6	MIBK^2^	0.50 g, 50.1%
		67.4% ee (*S*)
7	MTBE^2^	0.46 g, 46.1%
		42.7% ee (*S*)
8	Toluene	0.13 g, 13.2%
		88.0% ee (*S*)
9	MeCN	0.048 g, 4.8%
		36.3% ee (*S*)

^1^ Yield was calculated based on the molar number of amine obtained as the salt. ^2^ THF: tetrahydrofuran; IPE: diisopropyl ether; MIBK: 4-methyl-2-pentanone; MTBE: methyl *tert*-butyl ether.

**Table 3:**
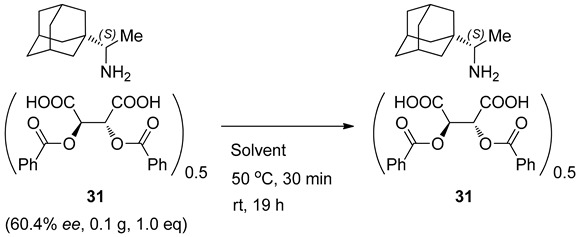
Screening of solvent for the second crystallization of diastereomerically enriched salt **31**.

Entry	Solvent	Results ^1^
1	Acetone (5% H_2_O)/60 v	0.084 g, 84%
		73.8% ee (S)
2	EtOAc (5% H_2_O)/60 v	0.090 g, 90%
		65.3% ee (S)
3	THF/30 v	0.036 g, 36%
		92.8% ee (*S*)

^1^ Yield was calculated based on the molar number of amine obtained as the salt.

**Table 4:**
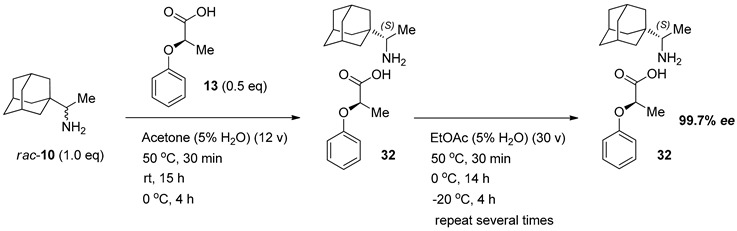
Application of (*R*)-phenoxypropionic acid **13** as a resolution reagent.

		3rd Batch (100 g)
1st salt formation	Yield	66.9 g, 34.7%
Acetone (5% H2O)	Optical purity ^1^	88.4% ee
2nd recrystallization	Yield	56.7 g, 87.3%
EtOAc (5% H2O)	Optical purity ^1^	98.7% ee
3rd recrystallization	Yield	51.1 g, 92.9%
EtOAc (5% H2O)	Optical purity ^1^	99.7% ee
Overall yield		28.1%

^1^ Optical purity was reconfirmed using the optimized HPLC method.

## Supplementary Materials

The following are available online: NMR spectra.
