# Supplementary material for: Optical Resolution of Rimantadine
Source: Molecules. 2019 May 12;24(9):1828. doi: 10.3390/molecules24091828 (PMC6539882; doi:10.3390/molecules24091828)
Supplement: Supplementary file 1 [file molecules-24-01828-s001.pdf]

Supporting Information

Optical Resolution of Rimantadine

Jianlin Han, Ryosuke Takeda*, Tatsunori Sato, Hiroki Moriwaki, Hidenori Abe, Kunisuke Izawa and Vadim A. Soloshonok*

1. **NMR spectra of rac-rimantadine 10………….……………………………………………2**

2. **Preparation of derivative 12 as a standard for chiroptical analysis…………………………3**

3. **NMR spectra for compounds 13-26……………………………………………………………5**

4. **Application of (R)-phenoxypropionic acid 13 as a resolution reagent………………………24**

1. NMR spectra of rac-rimantadine 10

^{1}H-NMR (200 MHz, $CDCl_3$) of *rac*-rimantadine **10**

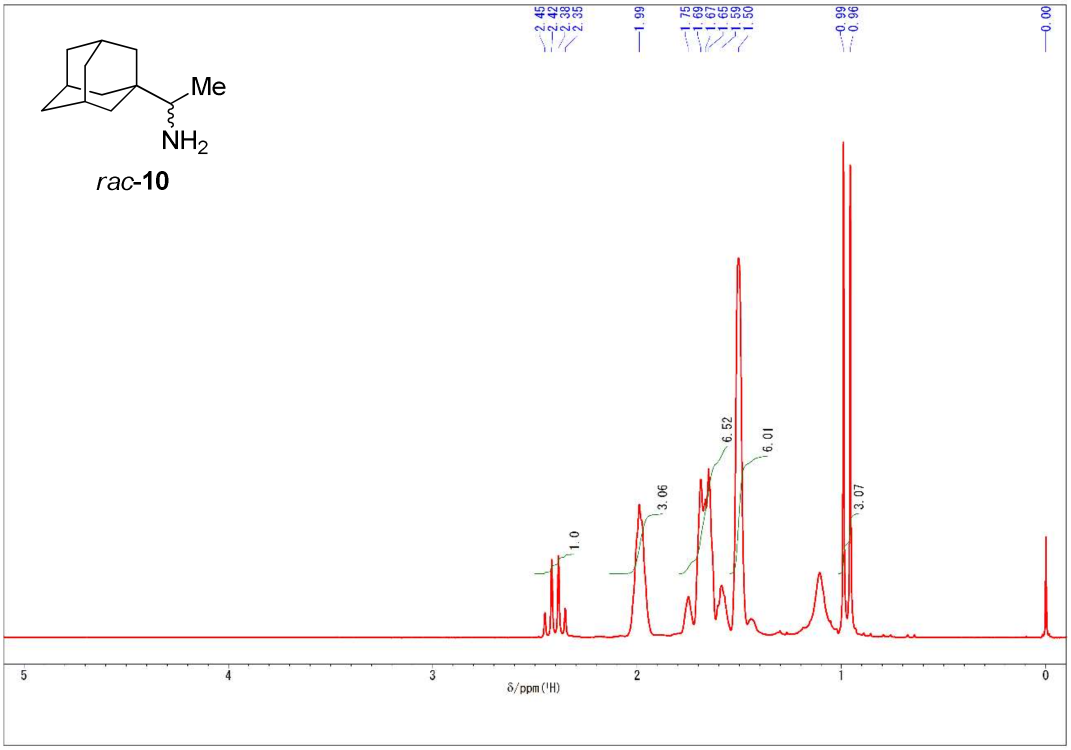

^{13}C-NMR (50 MHz, $CDCl_3$) of *rac*-rimantadine **10**

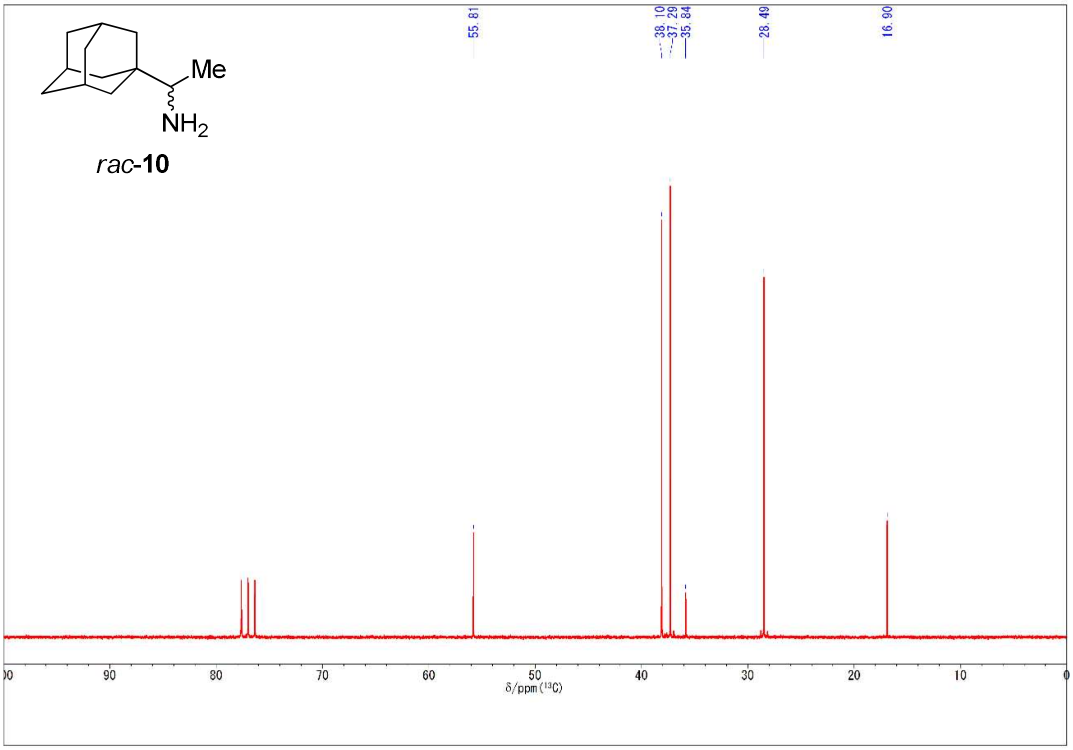

2. Preparation of derivative 12 as a standard for chiroptical analysis

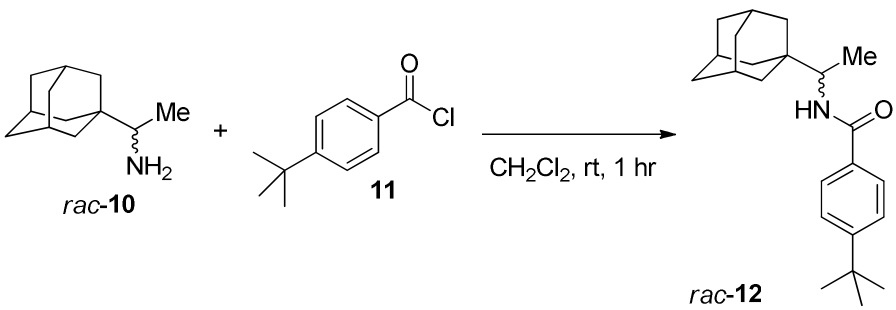

Scheme 3 (in the manuscript). Preparation of derivative **12** as a standard for chiroptical analysis.

To a mixture of *rac*-rimantadine **10** (25 mg, 0.14 mmol) in CH_2Cl_2 (3 mL) was added *p*-*tert*-Butylbenzoyl chloride (11 mg, 0.06 mmol). The reaction mixture was stirred at room temperature for 1 hour. The resultant mixture was evaporated at 40 °C and then dried under vacuum. The obtained residue was dissolved with IPA (5 mL). 2 mL of the prepared solution and 8 mL of hexane was mixed in 10 mL measuring flask and the sample was analysed by the following HPLC conditions.

Instrument: SHIMADZU LC-2010CHT chromatography system and a CLASS-VP™ analysis data system (SHIMADZU CORPORATION, Kyoto, Japan).
Column: DAICEL CHIRALPAK AD-H (5 µm, 250 x 4.6 mm i.d.)
Eluent: hexane: IPA: Et_2NH = 85: 15: 0.1
Flow rate: 1.0 mL/min
Temp: 25 ºC
Time: 15 min
Detector: UV 254 nm

Chiral HPLC chart of *rac*-**12**

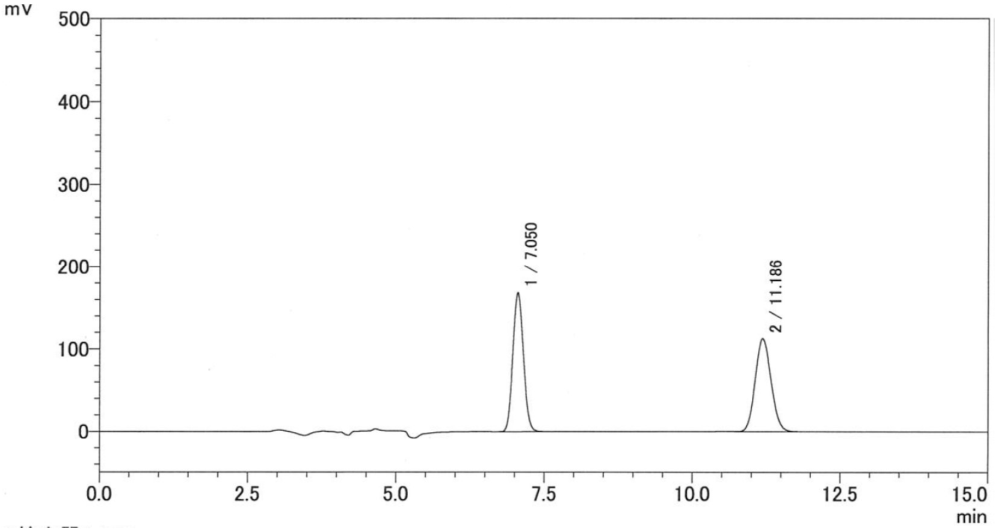

HPLC retention time (min)		Ratio ((*R*)-**12** : (*S*)-**12**)
(*R*)-**12**	(*S*)-**12**	
7.1	11.1	0.16% *ee* (49.9 : 50.1)

3. NMR spectra for compounds 13-26

#1 Rimantadine (10) (*R*)-2-Phenoxy propionic acid (13) 0.5 eq salt (99.7% *ee*)

51.1 g, 92.9%, 99.7% *ee* (*S*) from 55.0 g, 98.7% *ee* (*S*) salt, mp 167-171 °C. ^{1}H NMR (400 MHz, CD_3OD): δ = 7.15-7.29 (m, 2H), 6.80-6.92 (m, 3H), 4.40 (q, *J* = 6.7 Hz, 1H), 2.75 (q, *J* = 6.6 Hz, 1H), 1.96-2.09 (m, 3H), 1.55-1.86 (m, 12H), 1.42-1.52 (m, 4H), 1.15 (d, *J* = 6.6 Hz, 3H).

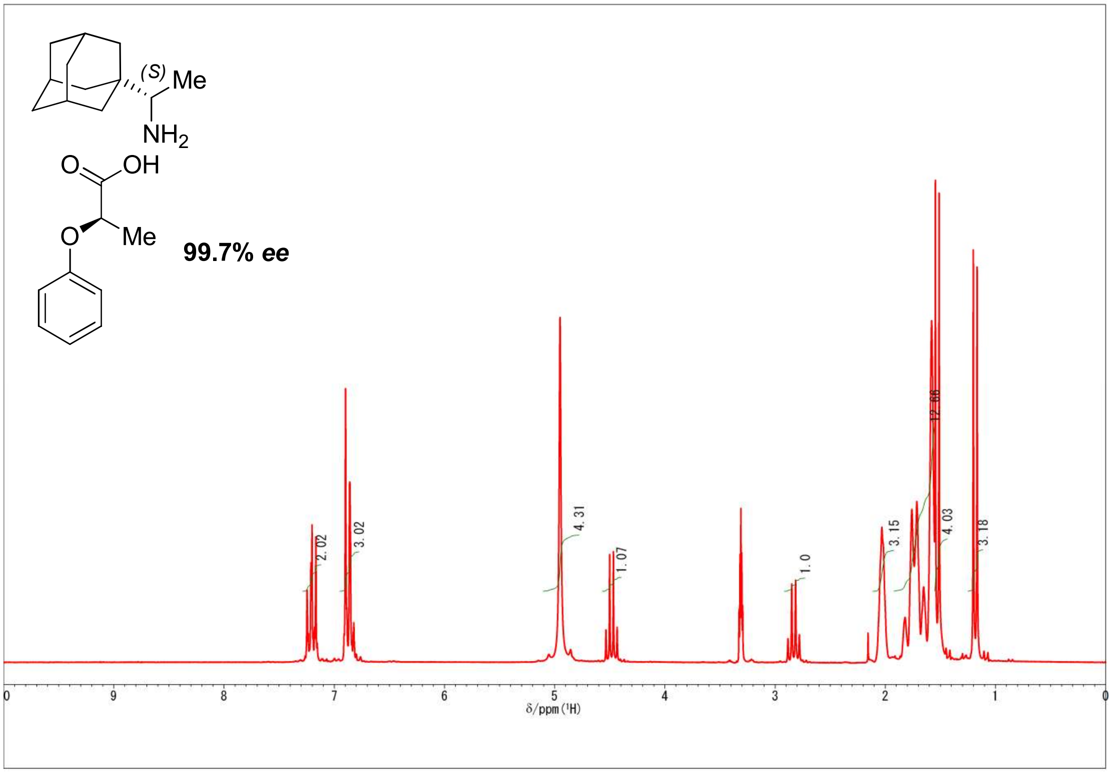

#2 Rimantadine (10) (*S*)-Naproxen (14) 0.5 eq. salt

0.51 g, 44.8% yield, 35.3% *ee* (*R*), mp 164-167 °C. ^{1}H NMR (400 MHz, CD_3OD): δ = 7.61-7.72 (m, 3H), 7.45-.7.55 (m, 1H), 7.15-7.20 (m, 1H), 7.02-7.11 (m, 1H), 3.85 (s, 3H), 3.61-3.79 (m, 1H), 3.65-3.85 (m, 1H), 1.95-2.05 (m, 3H), 1.50-1.85 (m, 12H), 1.45-1.49 (m, 3H), 1.11 (d, *J* = 6.6 Hz, 3H).

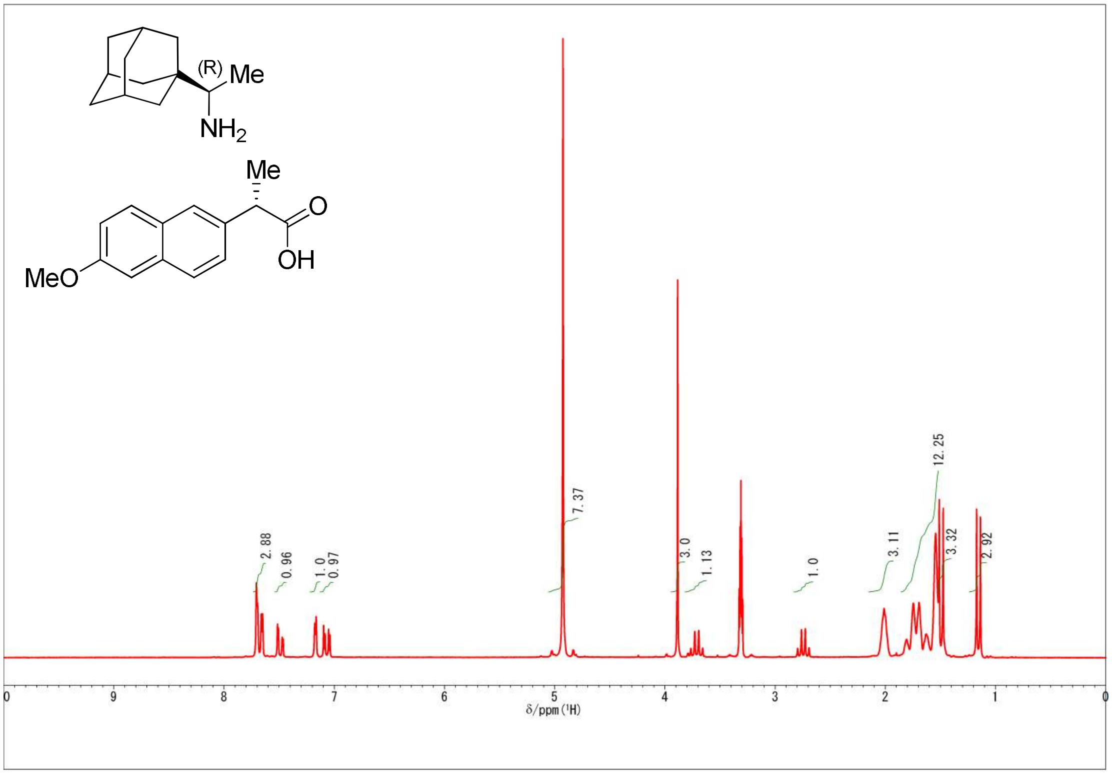

#3 Rimantadine (10) (*R*)-Mandelic acid (15) 0.5 eq salt

0.39 g, 42.0%, 7.32% *ee* (*S*), mp 154-156 °C. ^{1}H NMR (400 MHz, CD_3OD): δ = 7.40-7.55 (m, 2H), 7.16-.7.39 (m, 3H), 4.85 (s, 1H), 2.76 (q, *J* = 6.7 Hz, 1H), 1.97-2.09 (m, 3H), 1.42-1.89 (m, 13H), 1.13 (d, *J* = 6.7 Hz, 3H).

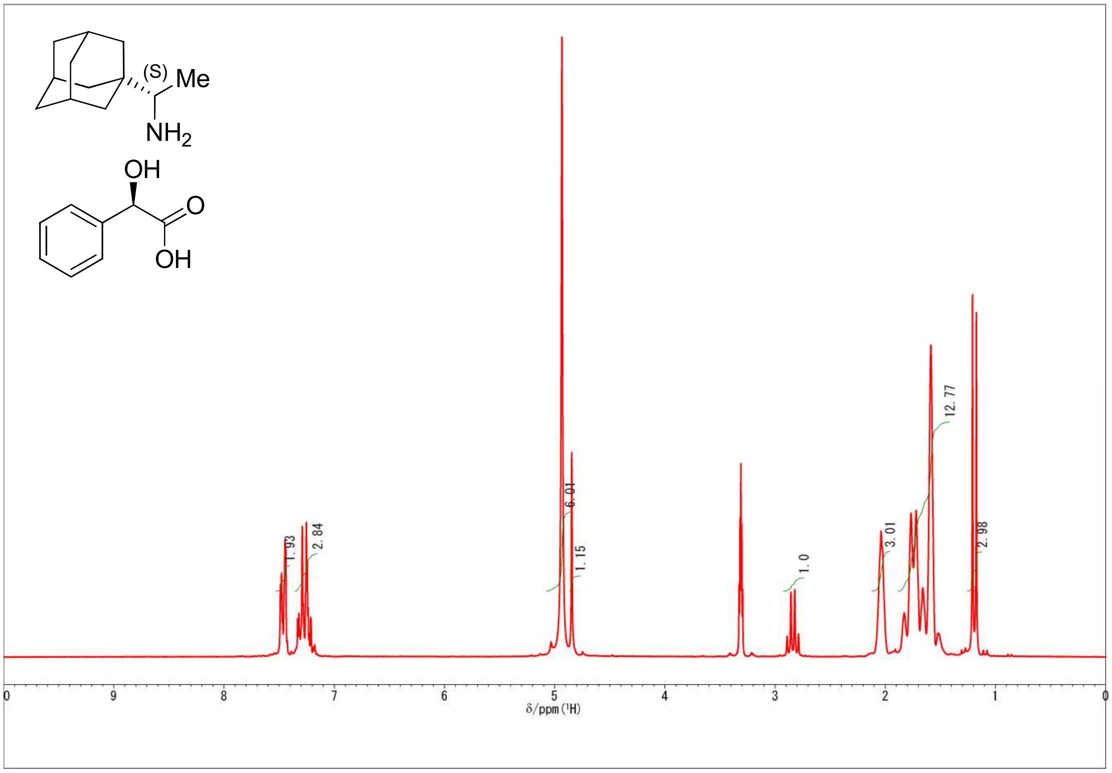

#4 Rimantadine (10) (*S*)-α-Methoxyphenylacetic acid (16) 0.5 eq salt

0.35 g, 36.1%, 1.34% *ee* (*S*), mp 178-181 °C. ^{1}H NMR (400 MHz, CD_3OD): δ = 7.45-7.55 (m, 2H), 7.21-.7.37 (m, 3H), 4.52 (s, 1H), 3.36 (s, 3H), 2.75 (q, *J* = 6.6 Hz, 1H), 1.95-2.06 (m, 3H), 1.42-1.89 (m, 13H), 1.13 (d, *J* = 6.6 Hz, 3H).

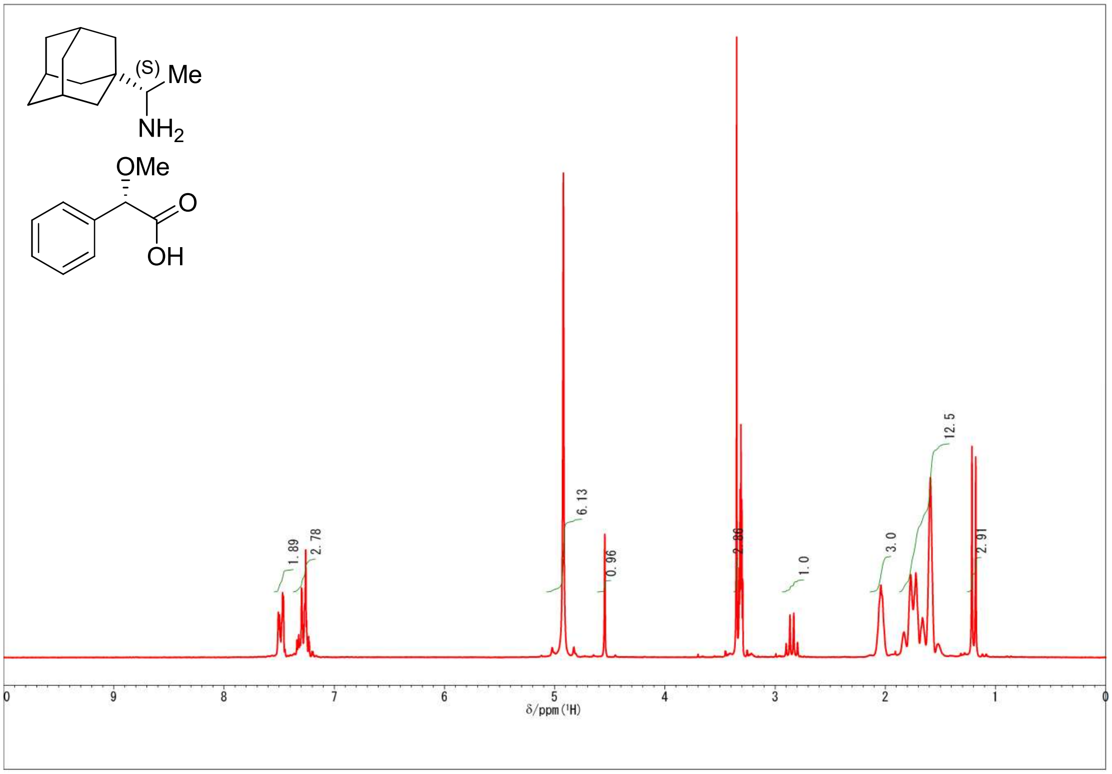

#5 Rimantadine (10) (*R*)-α-Methoxyphenylacetic acid (17) 0.5 eq salt

0.39 g, 40.1%, 0.08% *ee* (*S*), mp 177-181 °C. ^{1}H NMR (400 MHz, CD_3OD): δ = 7.45-7.55 (m, 2H), 7.20-.7.39 (m, 3H), 4.55 (s, 1H), 3.31 (s, 3H), 2.79 (q, *J* = 6.7 Hz, 1H), 1.95-2.09 (m, 3H), 1.49-1.85 (m, 13H), 1.15 (d, *J* = 6.6 Hz, 3H).

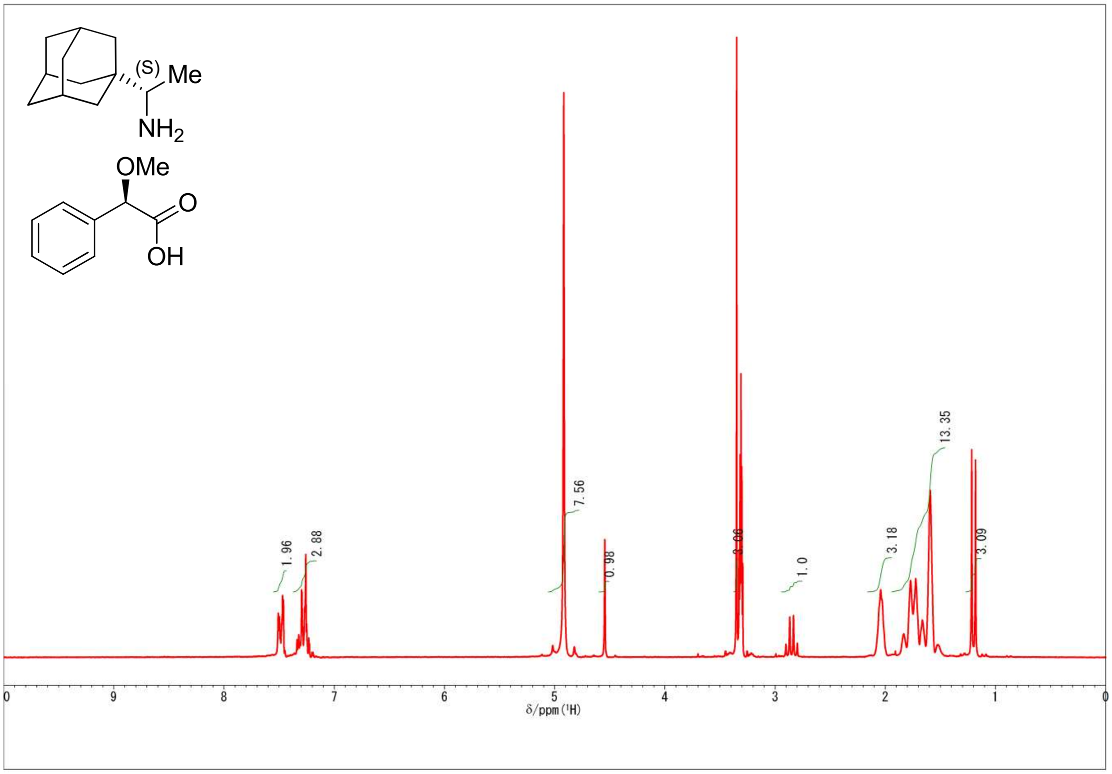

#6 Rimantadine (10) (*S*)-Aspartic acid (18) 0.5 eq salt

0.36 g, 52.0%, 7.44% *ee* (*S*), mp 205-208 °C. ^{1}H NMR (400 MHz, CD_3OD): δ = 4.69-4.79 (m, 1H), 2.75-2.93 (m, 2H), 2.49-2.63 (m, 1H), 1.98-2.09 (m, 3H), 1.45-1.90 (m, 13H), 1.18 (d, *J* = 6.6 Hz, 3H).

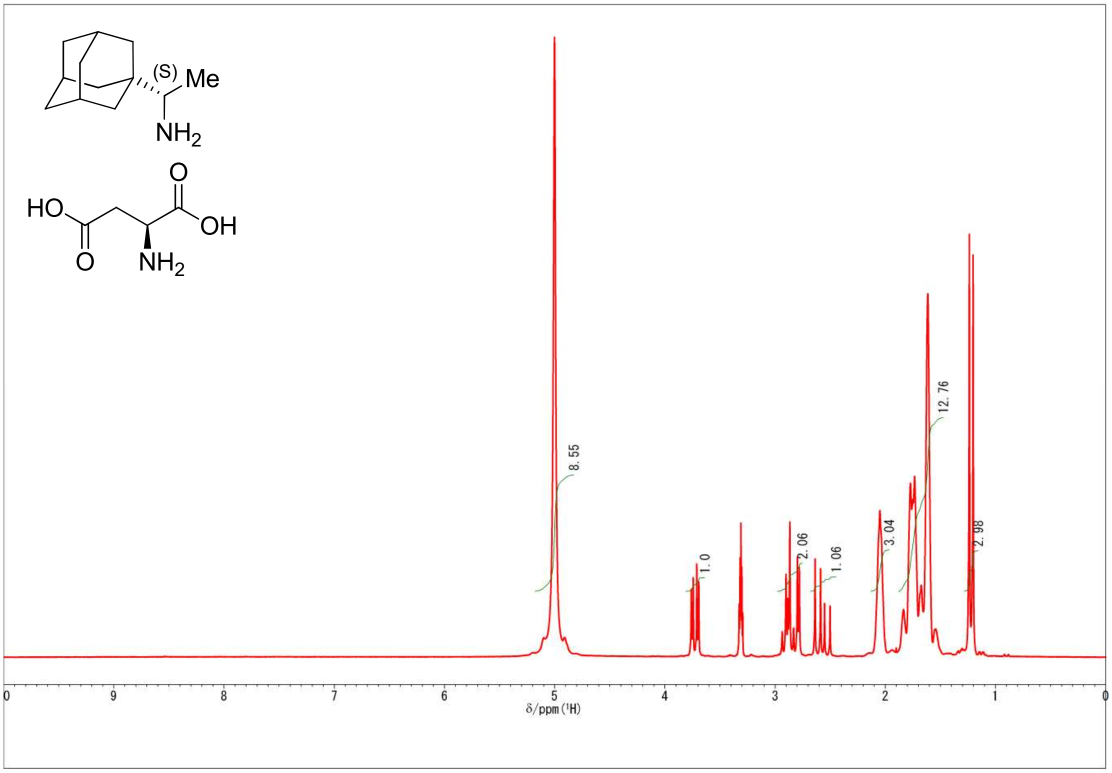

#7 Rimantadine (*S*)-Aspartic acid (18) 0.25 eq salt (1:1 salt)

0.17 g, 24.6%, 7.20% *ee* (*S*), mp 207-209 °C. ^{1}H NMR (400 MHz, CD_3OD): δ = 4.67-4.78 (m, 1H), 2.76-2.91 (m, 2H), 2.48-2.61 (m, 1H), 1.98-2.09 (m, 3H), 1.50-1.90 (m, 13H), 1.15 (d, *J* = 6.7 Hz, 3H).

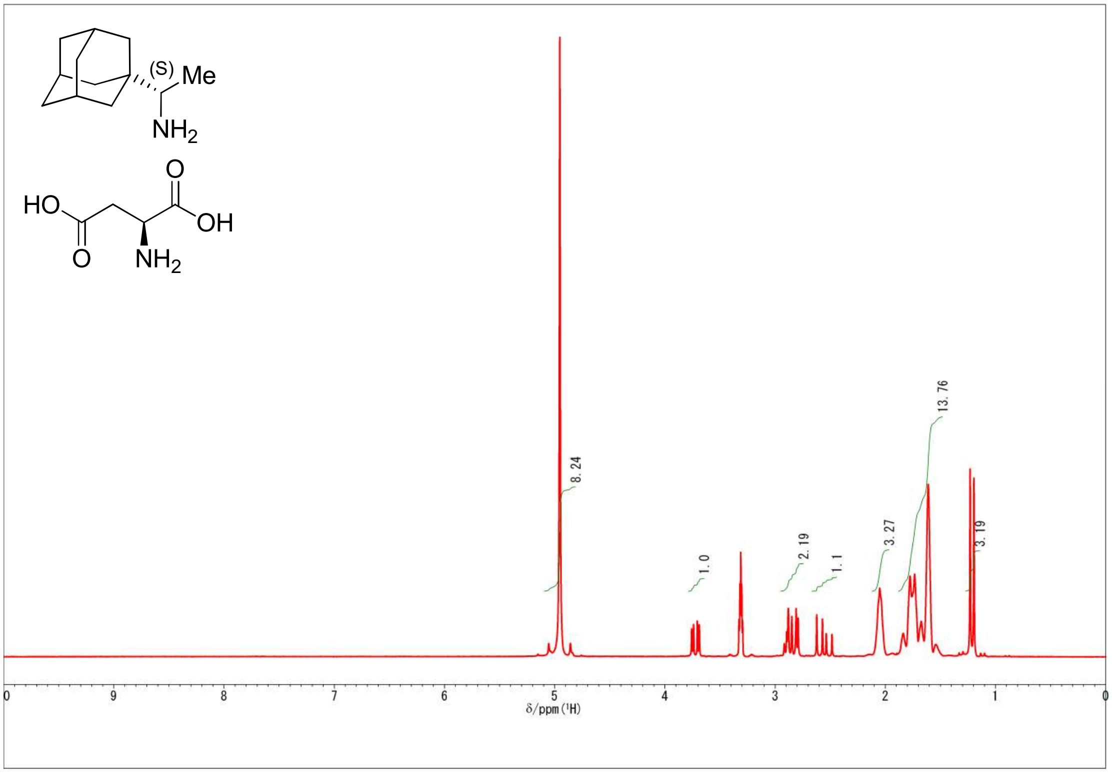

#8 Rimantadine (10) (*S*)-Malic acid (19) 1.0 eq salt

0.52 g, 59.2%, 0.20% *ee* (*S*), mp 206-209 °C. ^{1}H NMR (400 MHz, CD_3OD): δ = 4.21-4.31 (m, 1H), 2.80-2.92 (m, 2H), 2.65-2.79 (m, 1H), 2.35-2.51 (m, 1H), 1.98-2.12 (m, 6H), 1.49-1.89 (m, 27H), 1.18 (d, *J* = 6.7 Hz, 6H).

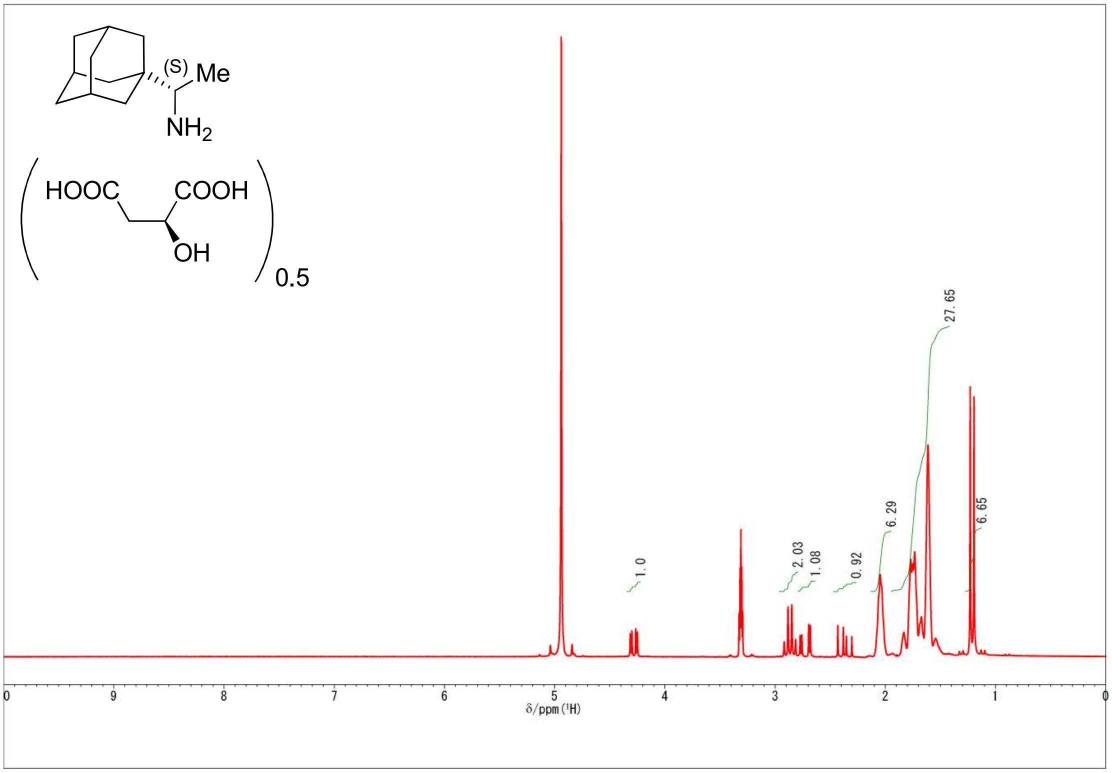

#9 Rimantadine (10) (*S*)-Malic acid (19) 0.25 eq salt

mp 207-211 °C. ^{1}H NMR (400 MHz, CD_3OD): δ = 4.21-4.31 (m, 1H), 2.78-2.92 (m, 2H), 2.65-2.79 (m, 1H), 2.39-2.51 (m, 1H), 1.97-2.10 (m, 6H), 1.51-1.88 (m, 26H), 1.18 (d, *J* = 6.7 Hz, 6H).

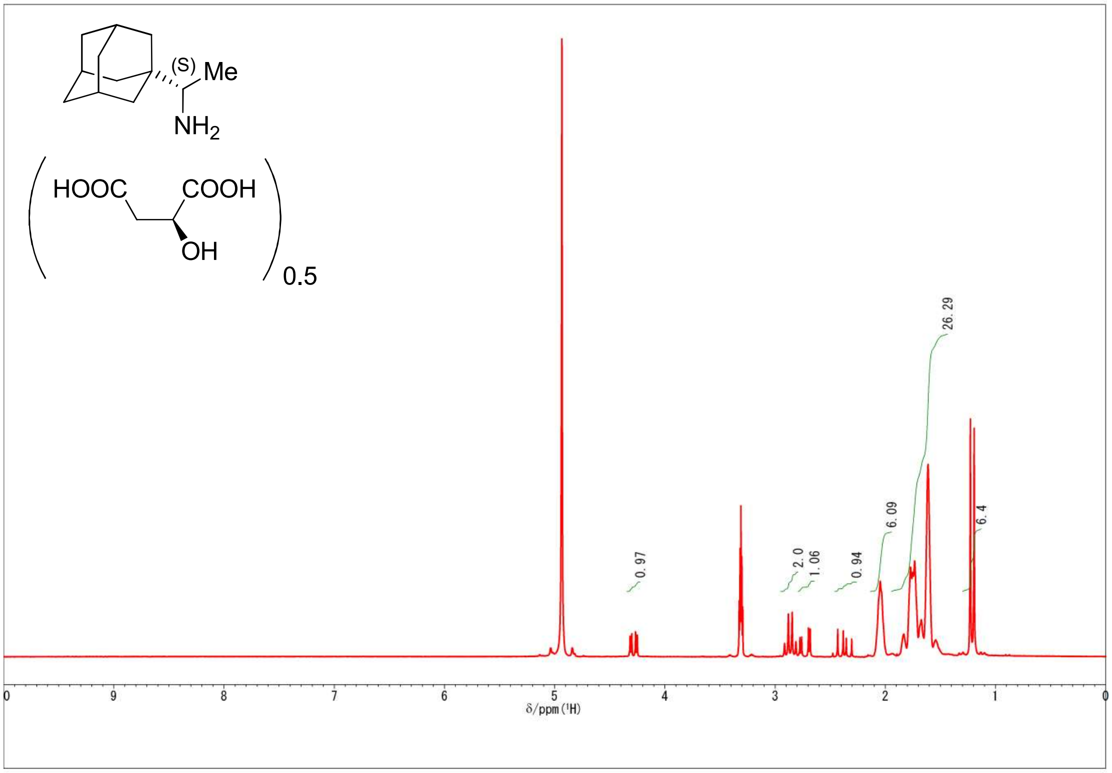

#10 Rimantadine (10) *N*-Tosyl-(S)-proline (20) 0.5 eq salt

0.08 g, 6.4%, 95.46% *ee* (*R*), mp 178-181 °C. ^{1}H NMR (400 MHz, CD_3OD): δ = 7.72-7.81 (m, 2H), 3.95-4.05 (m, 1H), 3.45-3.55 (m, 2H), 3.15-3.25 (m, 1H), 2.80-2.94 (m, 1H), 2.53 (s, 3H), 2.65-2.79 (m, 1H), 2.00-2.11 (m, 3H), 1.59-1.89 (m, 15H), 1.19 (d, *J* = 6.6 Hz, 3H).

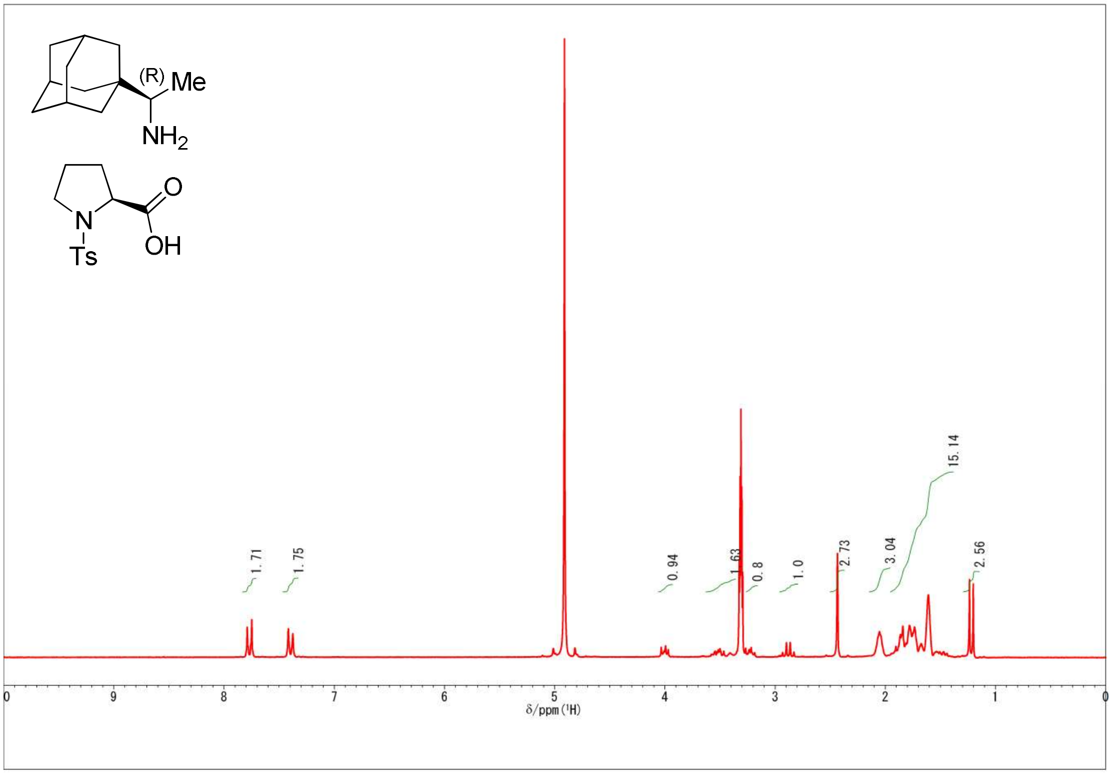

#11 Rimantadine (10) (1*R*,3*S*)-Camphoric acid (21) 0.5 eq salt

0.78 g, 100%, 0.72% *ee* (*S*), mp 180-182 °C. ^{1}H NMR (400 MHz, CD_3OD): δ = 2.70-2.90 (m, 1H), 2.42-2.70 (m, 1H), 1.98-2.11 (m, 3H), 1.60-1.88 (m, 14H), 1.45 (s, 1H), 1.35 (s, 3H), 1.18 (d, *J* = 6.6 Hz, 3H), 0.90 (s, 2H).

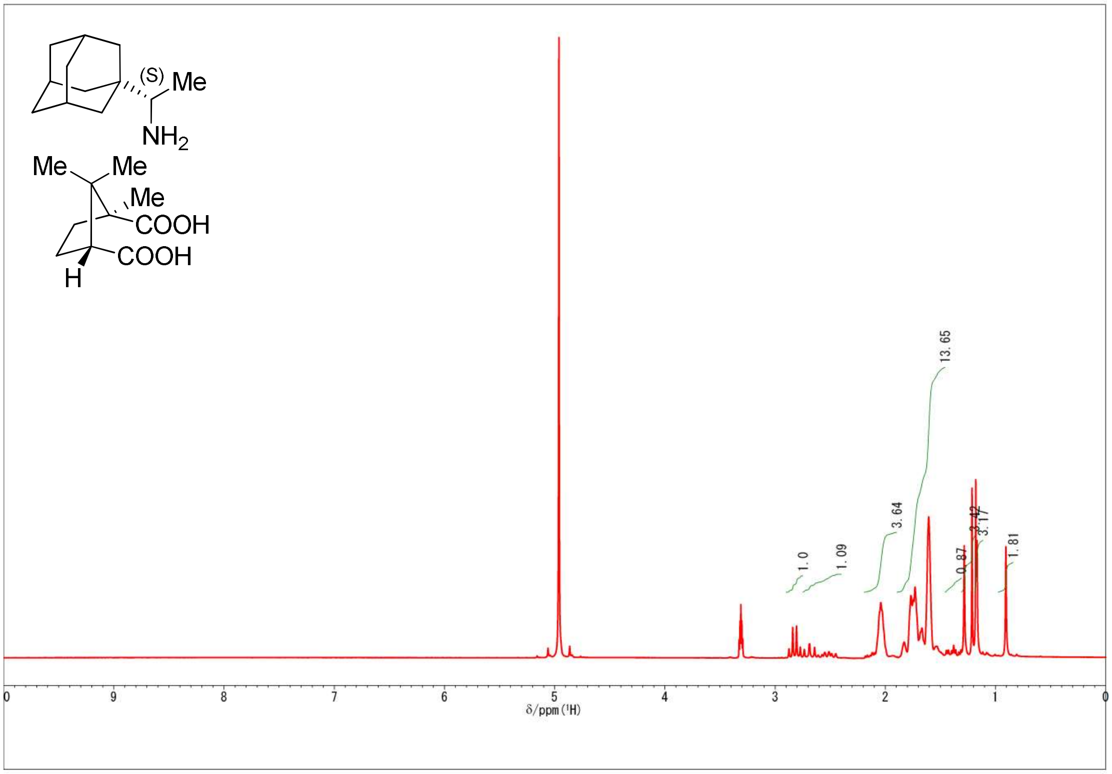

#12 Rimantadine (10) (1*R*,3*S*)-Camphoric acid (21) 0.25 eq salt

0.13 g, 16.8%, 0.38% *ee* (*S*), mp 182-186 °C. ^{1}H NMR (400 MHz, CD_3OD): δ = 2.70-2.85 (m, 1H), 2.52-2.70 (m, 1H), 1.97-2.12 (m, 3H), 1.55-1.88 (m, 12H), 1.38 (s, 1H), 1.14-1.22 (m, 6H), 0.90 (s, 1H).

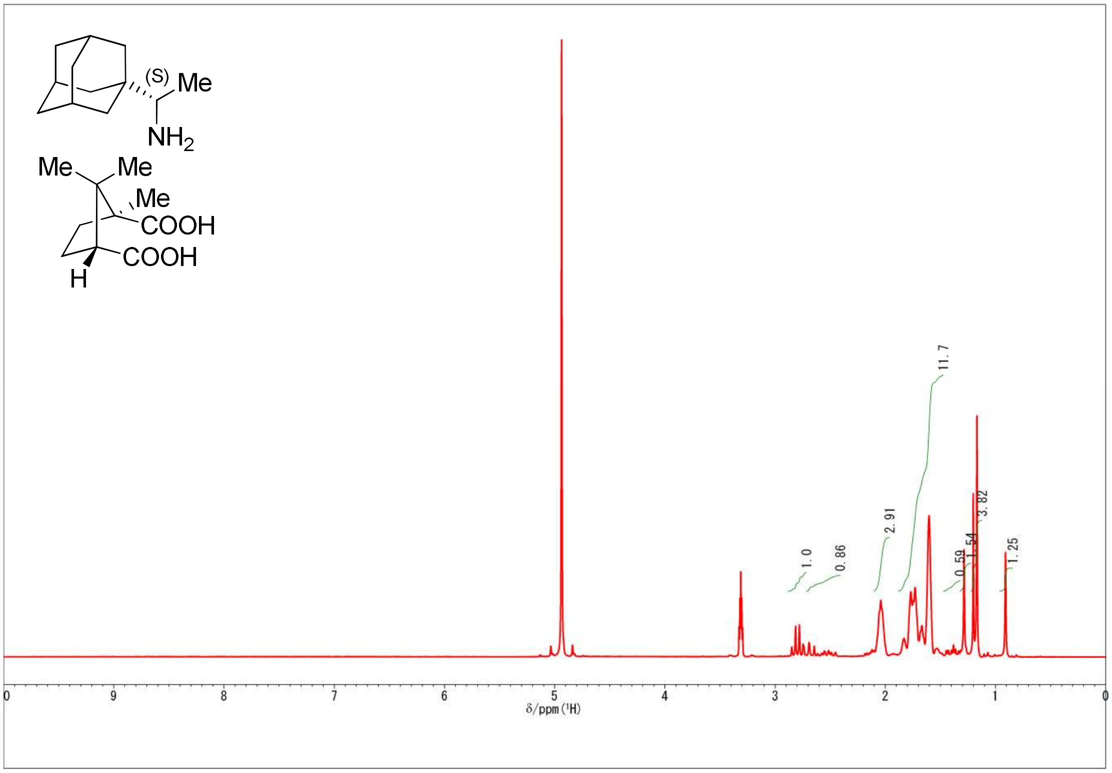

#13 Rimantadine (10) (1*S*)-10-Camphorsulforic acid (22) 1.0 eq salt

0.81 g, 70.9%, 5.22% *ee* (*R*), mp 204-208 °C. ^{1}H NMR (400 MHz, CD_3OD): δ = 3.31-3.41 (m, 2H), 2.82 (q, *J* = 6.5 Hz, 1H), 2.60-2.80 (m, 2H), 2.28-2.49 (m, 1H), 2.00-2.18 (m, 5H), 1.40-1.90 (m, 16H), 1.20 (d *J* = 6.5 Hz, 3H), 1.15 (s, 3H), 0.89 (s, 3H).

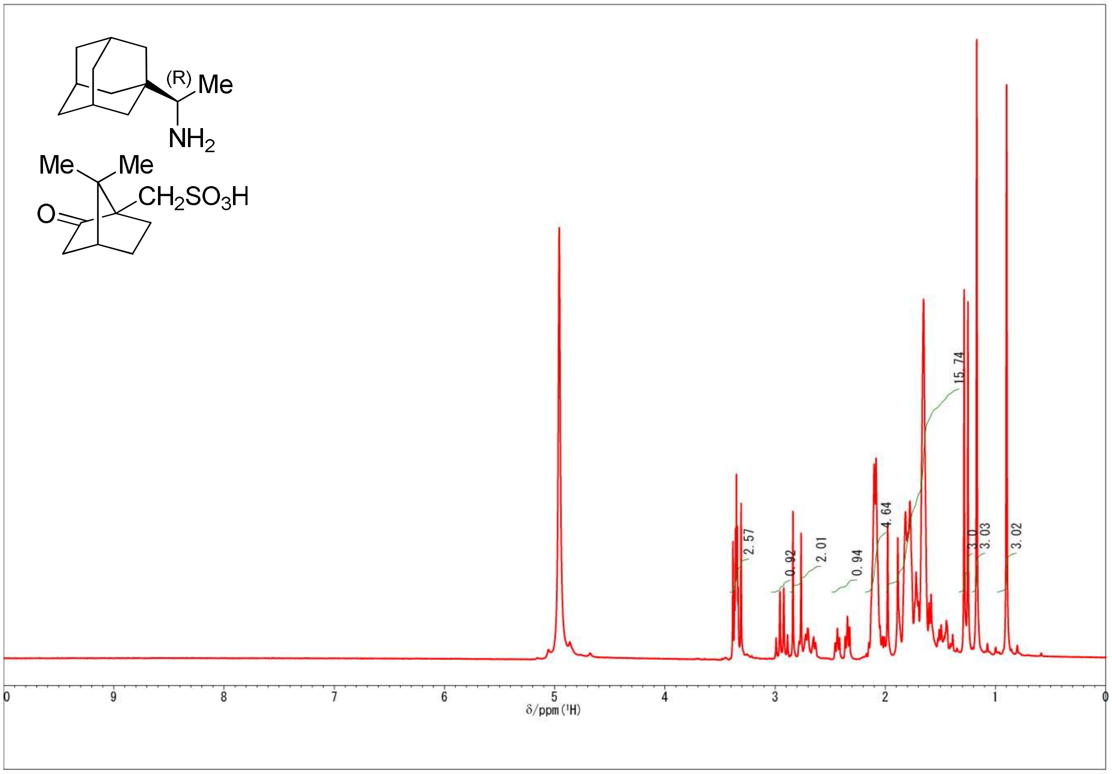

#14 Rimantadine (10) (2*S*,3*S*)-Tartaric acid (23) 0.5 eq salt

0.73 g, 103%, 1.38% *ee* (*S*), mp 220-221 °C. ^{1}H NMR (400 MHz, CD_3OD): δ = 4.30 (s, 1H), 2.80 (q, *J* = 6.5 Hz, 1H), 1.96-2.18 (m, 3H), 1.45-1.89 (m, 12H), 1.15 (d, *J* = 6.5 Hz, 3H).

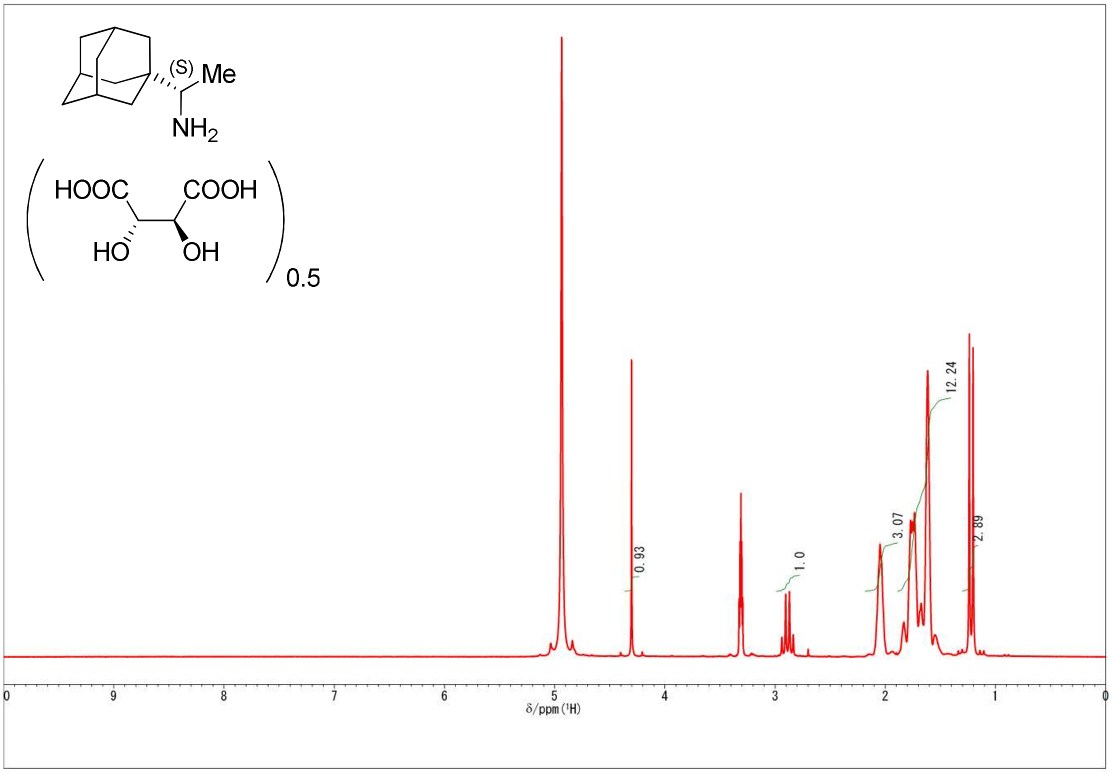

#15 Rimantadine (10) (2*S*,3*S*)-Tartaric acid (23) 0.25 eq salt

0.35 g, 49.0%, 23.24% *ee* (*S*), mp 217-219 °C. ^{1}H NMR (400 MHz, CD_3OD): δ = 4.30 (s, 1H), 2.81 (q, *J* = 6.6 Hz, 1H), 1.97-2.08 (m, 3H), 1.44-1.88 (m, 13H), 1.12 (d, *J* = 6.5 Hz, 3H).

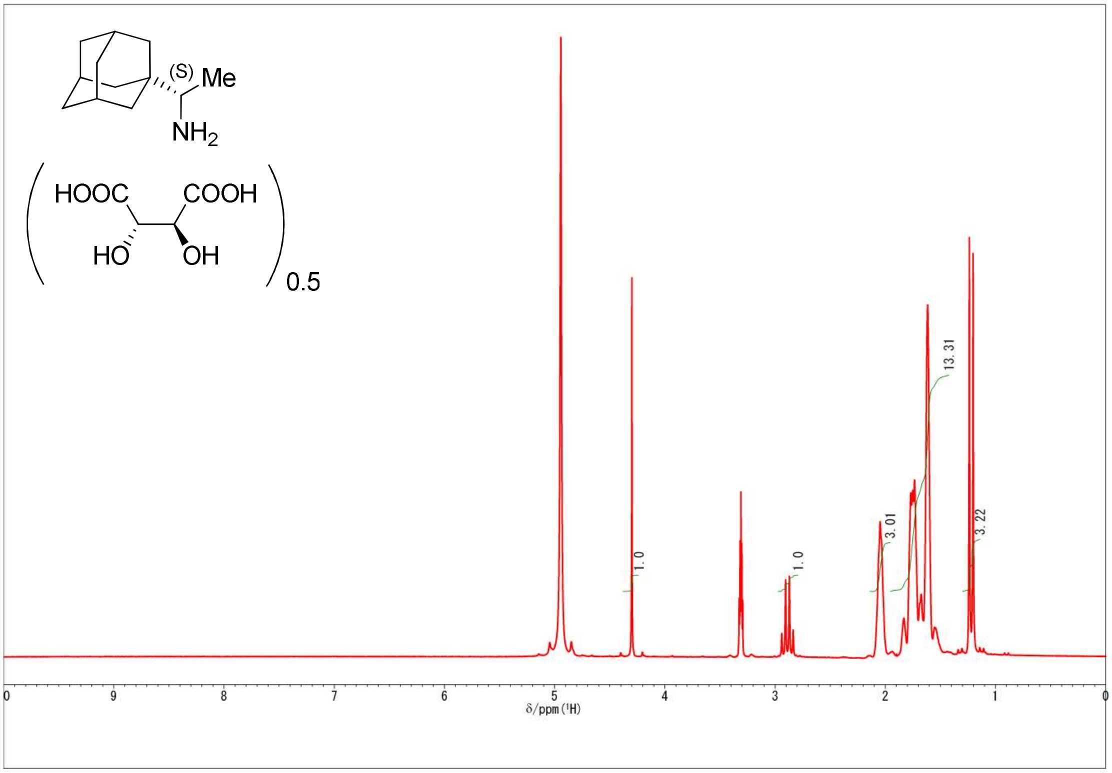

#16 Rimantadine (10) (2*S*,3*S*)-Dibenzoyltartaric acid (24) 0.5 eq salt (2:1 salt)

0.87 g, 86.6%, 16.38% *ee* (*R*), mp 168-171 °C. ^{1}H NMR (400 MHz, CD_3OD): δ = 8.15-8.22 (m, 2H), 7.50-7.75 (m, 3H), 5.89 (s, 1), 2.72 (q, J = 6.6 Hz, 1H), 1.91-2.08 (m, 3H), 1.42-1.89 (m, 12H), 1.11 (d, J = 6.5 Hz, 3H).

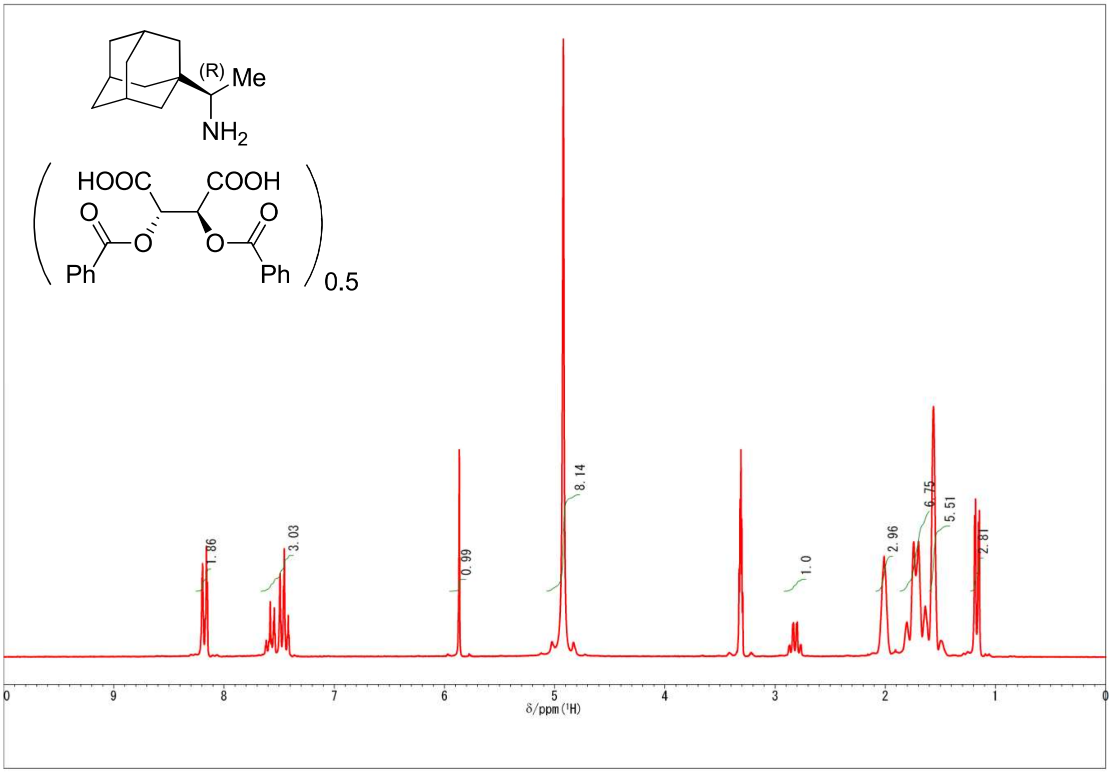

#17 Rimantadine (10) (2*S*,3*S*)-Dibenzoyltartaric acid (24) 0.25 eq salt

0.48 g, 48.4%, 62.72% *ee* (*R*), mp 178-179 °C. ^{1}H NMR (400 MHz, CD_3OD): δ = 8.12-8.22 (m, 2H), 7.40-7.75 (m, 3H), 5.86 (s, 1), 2.72 (q, *J* = 6.5 Hz, 1H), 1.92-2.08 (m, 3H), 1.40-1.85 (m, 12H), 1.10 (d, *J* = 6.5 Hz, 3H).

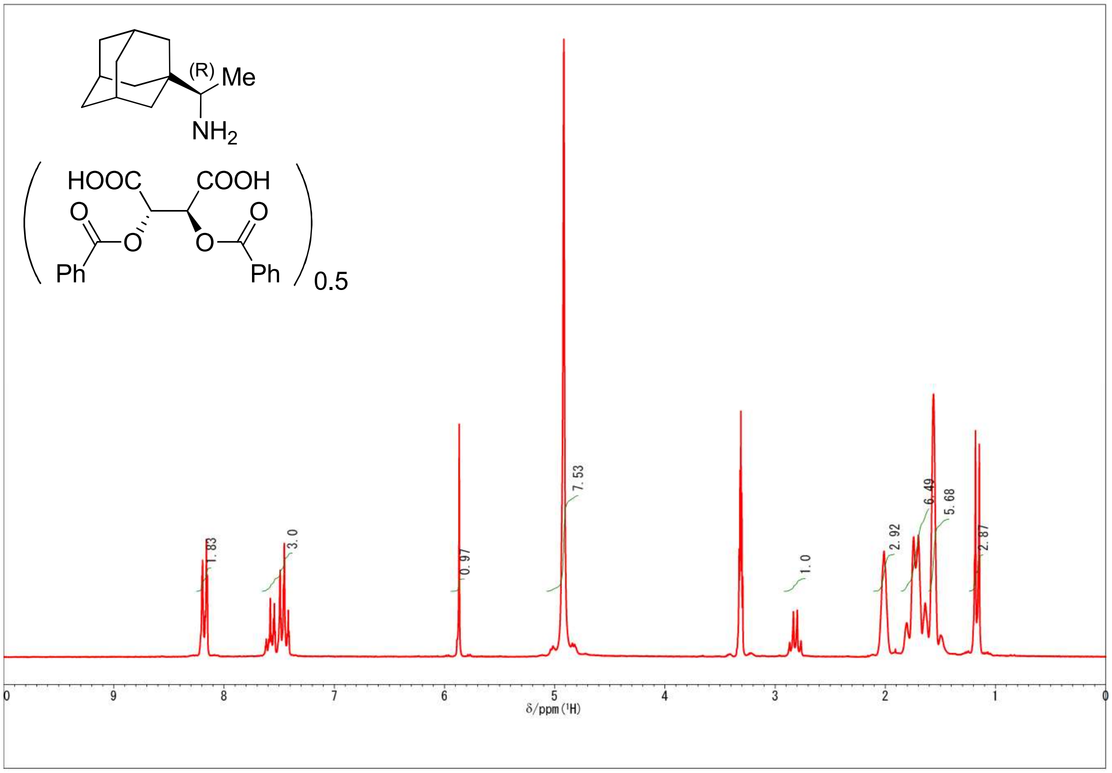

#18 Rimantadine (10) (2*R*,3*R*)-Dibenzoyltartaric acid monohydrate (25) 0.25 eq salt (94.8% *ee*)

0.242 g, 48.4%, 94.8% *ee* (*S*) from 0.50 g, 67.0% *ee* (*S*) salt, mp 177-179 °C. ^{1}H NMR (400 MHz, CD_3OD): δ = 8.13-8.22 (m, 2H), 7.50-7.77 (m, 3H), 5.87 (s, 1), 2.72 (q, *J* = 6.5 Hz, 1H), 1.92-2.12 (m, 3H), 1.40-1.85 (m, 12H), 1.11 (d, *J* = 6.5 Hz, 3H).

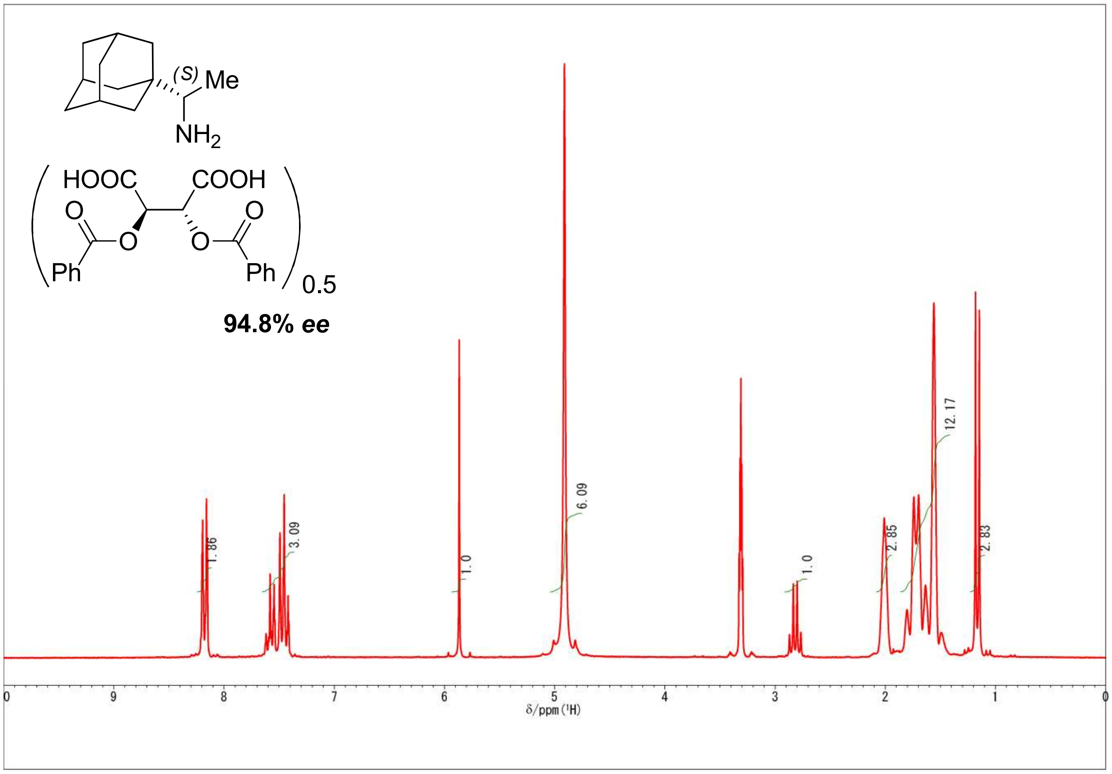

#19 Rimantadine (10) (2*R*,3*R*)-Di-p-toluoyltartaric acid (26) 1.0 eq salt

1.612 g, 102%, 0.38% *ee* (*R*), mp 215-216 °C. ^{1}H NMR (400 MHz, CD_3OD): δ = 7.97-8.05 (m, 4H), 7.25-7.35 (m, 4H), 5.87 (s, 1), 2.75-2.90 (m, 1H), 2.40 (s, 6H), 1.95-2.08 (m, 3H), 1.40-1.86 (m, 12H), 1.15 (d, *J* = 6.5 Hz, 3H).

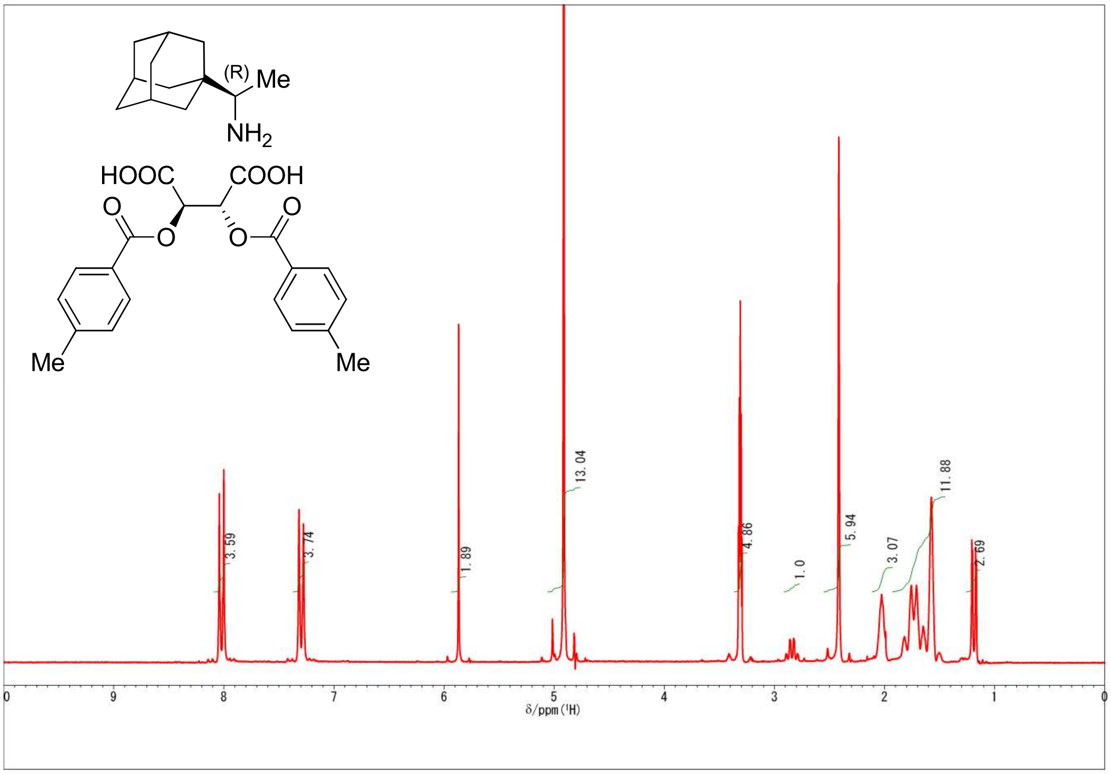

#20 Rimantadine (10) (2*R*,3*R*)-Di-p-toluoyltartaric acid (26) 0.25 eq salt

0.53 g, 50.6%, 14.12% *ee* (*S*), mp 177-180 °C. ^{1}H NMR (400 MHz, CD_3OD): δ = 8.01-8.09 (m, 2H), 7.20-7.31 (m, 2H), 5.82 (s, 1), 2.70-2.88 (m, 1H), 2.40 (s, 3H), 1.90-2.06 (m, 3H), 1.40-1.86 (m, 13H), 1.10 (d, *J* = 6.5 Hz, 3H).

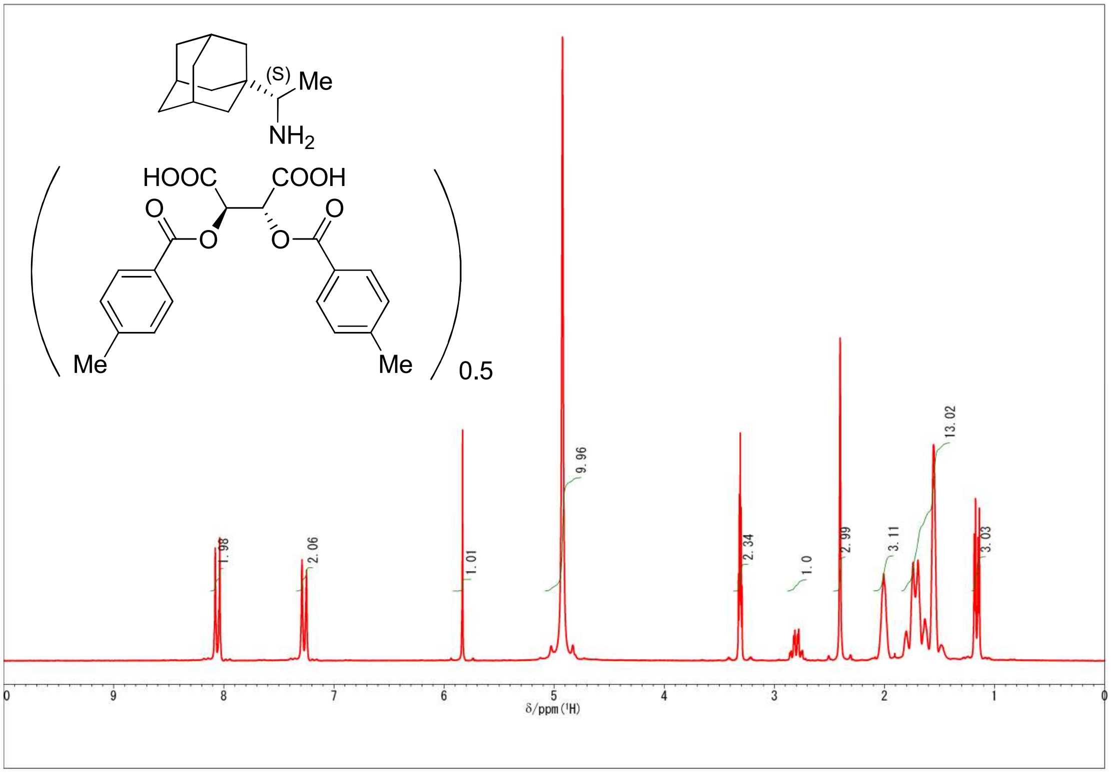

4. Application of (R)-phenoxypropionic acid 13 as a resolution reagent

To the solution of *rac*-Rimantadine **10** (100 g, 558 mmol) in acetone with 5% H_2O (600 mL) was added (*R*)-2- phenoxypropionic acid **13** (46.3 g, 279 mmol) in acetone with 5% H_2O (600 mL). The mixture was stirred for 30 minutes at 50°C, then cooled to room temperature and stirred for 15 hours to form precipitate. After that the suspension was cooled to 0 ^{o}C and stirred for 4 hours. The precipitate was filtered, washed with acetone with 5% H_2O and dried under vacuum at 40 ^{o}C to afford 1st salt **32** (66.9 g, 34.7%, 88.4% *ee*).

The mixture of 65 g of obtained 1st salt **32** in EtOAc with 5% H_2O (1950 mL) was stirred at 50 ^{o}C for 30 minutes until the salt dissolved, then cooled to 0 ^{o}C and stirred for 14 hours to form precipitate. After that the suspension was cooled to -20 ^{o}C and stirred for 4 hours. The precipitate was filtered, washed with EtOAc and dried under vacuum at 40 ^{o}C to afford 2nd salt **32** (56.7 g, 87.3%, 98.7% *ee*).

The mixture of 55 g of obtained 2nd salt **32** in EtOAc with 5% H_2O (1650 mL) was stirred at 50 ^{o}C for 30 minutes until the salt dissolved, then cooled to 0 ^{o}C and stirred for 14 hours to form precipitate. After that the suspension was cooled to -20 ^{o}C and stirred for 4 hours. The precipitate was filtered, washed with EtOAc and dried under vacuum at 40 ^{o}C to afford 3rd salt **32** (51.1 g, 92.9%, 99.7% *ee*).

Chiral HPLC chart of (*S*)-**12** prepared from 1st salt **32**

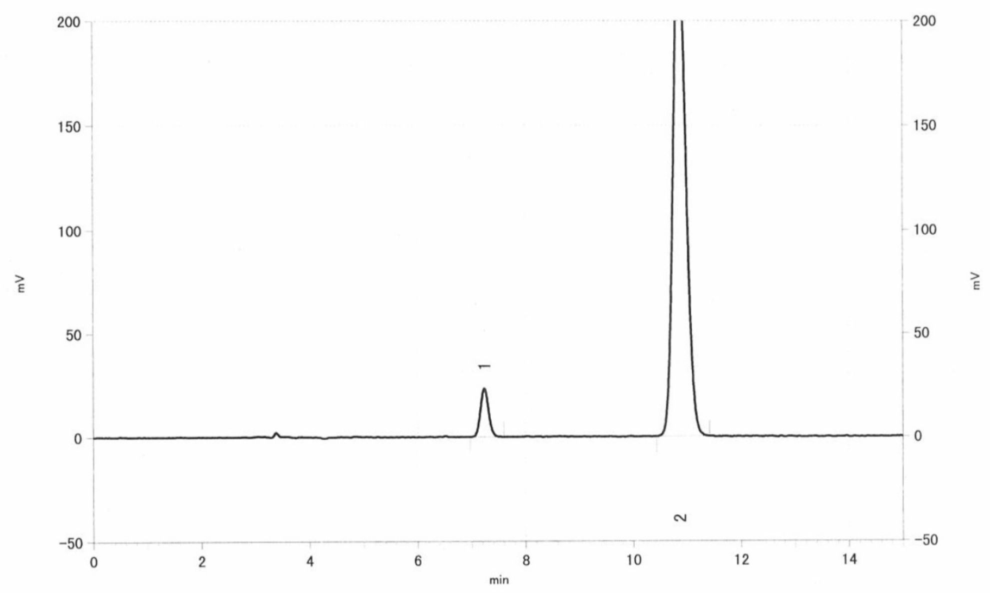

HPLC retention time (min)		Ratio ((*R*)-**12** : (*S*)-**12**)
(*R*)-**12**	(*S*)-**12**	
7.1	11.1	88.4% *ee* (5.8 : 94.2)

Chiral HPLC chart of (*S*)-**12** prepared from 2nd salt **32**

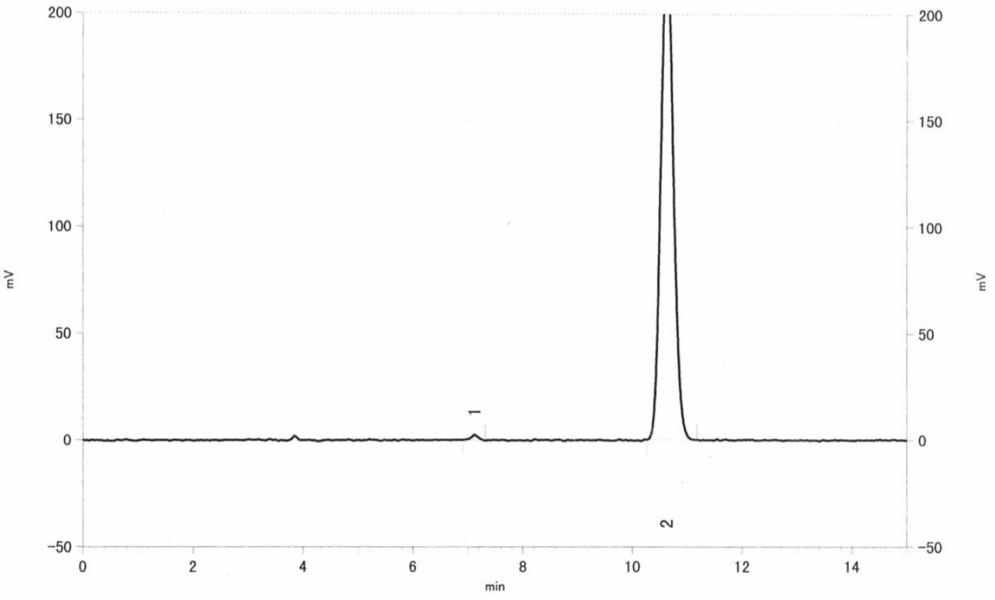

HPLC retention time (min)		Ratio ((*R*)-**12** : (*S*)-**12**)
(*R*)-**12**	(*S*)-**12**	
7.1	11.1	98.7% *ee* (0.7 : 99.3)

Chiral HPLC chart of (*S*)-**12** prepared from 3rd salt **32**

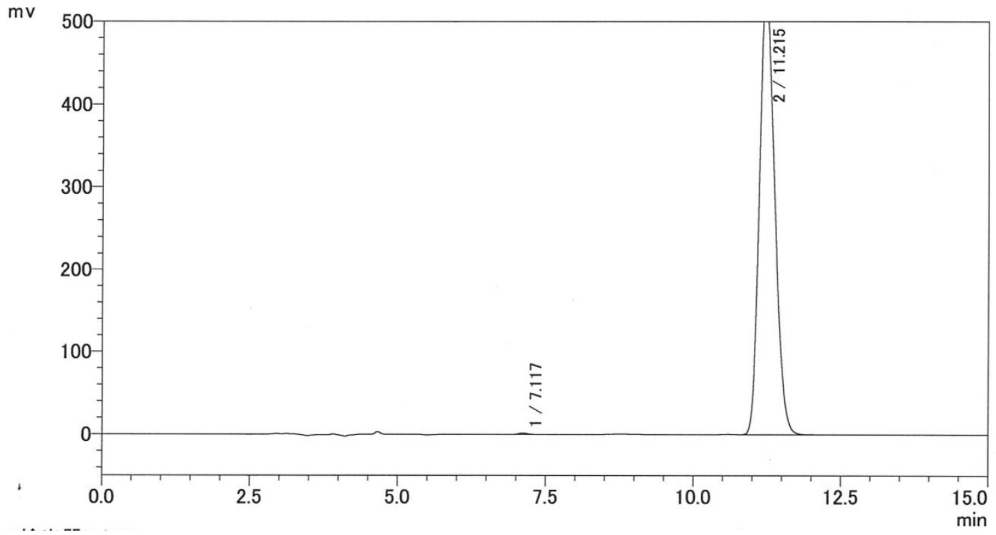

HPLC retention time (min)		Ratio ((*R*)-**12** : (*S*)-**12**)
(*R*)-**12**	(*S*)-**12**	
7.1	11.2	99.7% *ee* (0.2 : 99.8)
